# The Two Faces of Copper Capture Driven by the Internal His/Met-Rich and N‑Terminal His-Rich Domains of CopI

**DOI:** 10.1021/acs.inorgchem.6c00726

**Published:** 2026-04-24

**Authors:** Paulina Sobol, Arian Kola, Daniela Valensin, Aleksandra Hecel

**Affiliations:** † Faculty of Chemistry, 49572 University of Wroclaw, 50383 Wroclaw, Poland; ‡ Department of Biotechnology, Chemistry and Pharmacy, 9313 University of Siena, Siena 53100, Italy; § Consorzio Interuniversitario Risonanze Magnetiche di Metalloproteine (CIRMMP), Via L. Sacconi 6, Sesto Fiorentino 50019, Italy

## Abstract

While copper is an essential redox cofactor, excess copper, particularly Cu­(I), is highly toxic and therefore requires precise control of metal speciation in the bacterial periplasm. CopI is a cupredoxin-like protein implicated in copper tolerance under microaerobic conditions and contains multiple histidine- and methionine-rich regions, yet its metal-binding properties remain poorly understood. Here, we deconstruct*Rubrivivax gelatinosus*CopI into its constituent metal-binding modules and provide the first quantitative, domain-resolved analysis of their Cu­(II) coordination chemistry. Two peptide domains, an N-terminal His-rich region and an internal His/Met-rich motif, form mononuclear 1:1 Cu­(II) complexes but exhibit strikingly different binding modes. The His-rich domain displays higher Cu­(II) affinity and a rigid, predominantly nitrogen-based coordination environment, whereas the His/Met-rich motif binds Cu­(II) more weakly and adopts a heterogeneous, N/S/O-donor coordination sphere indicative of structural flexibility. Neither domain forms stable secondary structure upon metal binding, indicating that intrinsic disorder is a functional feature rather than a structural deficiency. Comparative Zn­(II) studies reveal substantially weaker binding, underscoring copper selectivity. Collectively, these results identify CopI as a chemically modular copper buffer and establish a coordination-chemical framework for understanding copper handling by periplasmic cupredoxin-like proteins.

## Graphical abstract

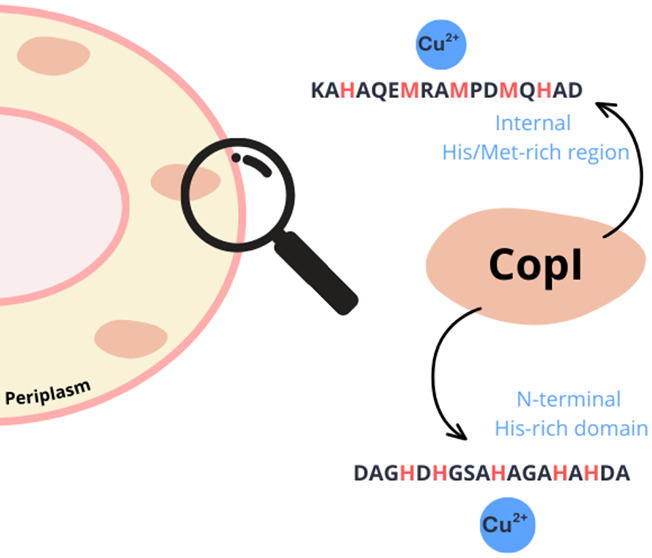

## Introduction

Copper is an essential cofactor in many biological redox processes, yet it becomes highly toxic when present in excess, especially in its reduced Cu­(I) form.^1–3^ Gram-negative bacteria therefore rely on a set of periplasmic proteins that bind, redistribute, or oxidize copper to protect the cell envelope.^4,5^ Disturbances in this network strongly affect bacterial viability and pathogenicity, as copper plays a dual role as a nutrient and a component of the innate immune response.^6,7^ Increasing environmental accumulation of copper and the growing interest in exploiting its antimicrobial properties have therefore intensified the need to understand the molecular mechanisms governing bacterial copper tolerance.^8,9^ Because copper homeostasis plays a central role in bacterial physiology, clarifying its mechanisms could help identify novel antibacterial targets, a possibility highlighted by recent findings on copper-export machinery.^10–12^

In addition to the well-known CueO/Cus systems, recent work has revealed the existence of a distinct group of cupredoxin-like proteins involved in copper tolerance under low-oxygen conditions.^13^ One representative of this group is CopI, identified in *Rubrivivax gelatinosus* and several pathogens such as*Vibrio cholerae*and*Pseudomonas aeruginosa*. Its physiological role becomes particularly important under microaerobic or anaerobic growth conditions, where Cu­(I) is the predominant copper species and must be tightly controlled by bacterial copper homeostasis systems.^5,14^ Within the framework of the Pearson hard–soft acid–base (HSAB) concept, Cu­(I) is classified as a soft Lewis acid and therefore preferentially interacts with soft sulfur donors such as the thiolate groups of cysteine and the thioether sulfur of methionine. CopI is unusual for a single-domain cupredoxin. In addition to the (i) classical His-Cys-His-Met motif that forms a green type-1.5 copper site (2N2S), the protein contains two extra segments enriched in histidines and methionines: (ii) a high-affinity N-terminal His-rich site and (iii) a third site located within the internal His/Met-rich region. Notably, the internal segment (residues 83–95 in*R. gelatinosus*CopI) is highly conserved among CopI homologues and is crucial for copper resistance, whereas the N-terminal His-rich region shows greater variability across species but frequently contributes an additional Cu­(II)-binding site.^13,14^ Spectroscopic studies of the full-length CopI protein and its mutant variants have suggested that the protein may accommodate several copper ions distributed between the cupredoxin center and additional peripheral binding regions. In particular, EPR analyses have proposed that the N-terminal site may coordinate Cu­(II) through predominantly nitrogen-based ligands (2N_im_, 1NH_2_, 1O), whereas the conserved His/Met-rich domain appears to participate in copper handling through a more flexible environment involving histidine and methionine residues. These observations are consistent with a functional model in which CopI binds Cu­(I) within the flexible His/Met-rich region and subsequently transfers the metal to the cupredoxin center for oxidation to Cu­(II), followed by redistribution of the oxidized metal among alternative binding sites.^8^

Despite these advances, the intrinsic coordination chemistry of individual CopI metal-binding motifs has never been quantified. Full-length CopI is a relatively large, multisite metalloprotein with significant conformational flexibility in the His/Met-rich segments, which complicates direct mechanistic dissection. As a result, it remains unclear whether the individual His-rich and His/Met-rich regions represent redundant copper-binding sites or chemically specialized modules with distinct coordination strategies. To address this challenge, we adopt a reductionist approach based on peptide models designed to mimic two distinct domains of the CopI protein. By isolating these regions from the structural complexity of the full-length protein, this strategy enables direct characterization of their intrinsic coordination chemistry and functional differentiation. In this way, the peptide systems do not merely represent simplified fragments, but provide a focused framework to dissect how individual domains contribute to the overall copper-handling properties of CopI. Despite growing interest in CopI as a copper tolerance factor, its individual metal-binding motifs have never been examined from a coordination-chemical perspective. In particular, the intrinsic Cu­(II) binding properties, metal selectivity, and structural responses of the His-rich and His/Met-rich regions remain completely unexplored. As a consequence, it is currently unclear how CopI distributes copper among its multiple sites, or how these regions collectively support copper detoxification under microaerobic conditions. Although the internal His/Met-rich region of CopI is expected to preferentially bind Cu­(I), investigation of its Cu­(II) coordination chemistry is essential to define its intrinsic ligand preferences and to benchmark its behavior against the more rigid His-rich domain. The ability of CopI domains to coordinate Cu­(II) is directly relevant in the periplasmic environment, where fluctuations in oxygen availability and redox potential enable interconversion between Cu­(I) and Cu­(II).

To date, no study has addressed the intrinsic Cu­(II) coordination preferences, metal selectivity, and structural responses of individual CopI domains from a coordination-chemical perspective. In this work, we investigate two synthetic peptides derived from the*R. gelatinosus*CopI sequence: (1) **Ac-KAHAQEMRAMPDMQHAD-NH**_**2**_, representing the internal His/Met-rich Cu­(I)/Cu­(II)-binding motif and (2) **Ac-DAGHDHGSAHAGAHAHDA-NH**_**2**_, corresponding to the N-terminal His-rich region. The use of peptide models is a widely applied strategy in bioinorganic chemistry to dissect complex metalloproteins into individual metal-binding motifs that can be studied independently. Such an approach allows the intrinsic coordination properties of specific sequence fragments to be examined using techniques particularly well suited for small, well-defined systems, such as potentiometry and detailed pH-dependent spectroscopic analyses. At the same time, it should be noted that the coordination environment in the intact protein may be further influenced by tertiary structure and long-range interactions within the cupredoxin fold. Beyond their relevance for understanding CopI itself, studies of isolated metal-binding motifs can also provide general insights useful for the design of biomimetic metal-binding systems and for strategies targeting bacterial copper homeostasis. In this context, the two peptide systems should be considered minimal functional models of distinct CopI domains, enabling selective investigation of their intrinsic coordination properties.

Using a combination of potentiometry, ESI-MS, UV–vis spectroscopy, CD, EPR and NMR, we characterize the coordination chemistry of Cu­(II) with these peptides and map the structural features that govern metal specificity and redox-linked conformational changes. By comparing the intrinsic properties of the isolated motifs, we provide new molecular insight into how CopI partitions copper between its multiple binding regions and how the modular His/Met-rich elements contribute to selective Cu­(I)/Cu­(II) handling in the absence of the cupredoxin fold. Although the His/Met-rich region of CopI is often associated with Cu­(I) binding in the protein context, direct investigation of Cu­(I) binding was not pursued here because the experimental strategy used in this work, particularly potentiometric titrations, is not suitable for reliable thermodynamic characterization of Cu­(I) complexes in methionine-containing systems. In contrast to cysteine, methionine coordinates Cu­(I) through a thioether sulfur that is not associated with a protonation/deprotonation equilibrium, which precludes determination of stability constants by potentiometry.

These results advance the understanding of the sequence-structure–function relationships that underline CopI-mediated copper detoxification and offer a foundation for deciphering the molecular logic of a unique multisite green cupredoxin involved in bacterial copper tolerance.

## Experimental Section

### Materials

The peptides (**Ac-KAHAQEMRAMPDMQHAD-NH**_**2**_ **and Ac-DAGHDHGSAHAGAHAHDA-NH**_**2**_) were purchased from Karebay Biochem (certified purity: 98%) and were used as received. The samples for electrospray ionization mass spectrometry (ESI-MS) were prepared in extra pure methanol (Sigma-Aldrich)-water mixture. Cu­(II) and Zn­(II) perchlorates were high-purity products [Cu­(ClO_4_)_2_·6H_2_O from Sigma-Aldrich; Zn­(ClO_4_)_2_·6H_2_O from POCH]. The concentrations of their stock solutions were determined by inductively coupled plasma mass spectrometry. The 0.1 M NaOH solution (Sigma-Aldrich), which was free of carbonates, was standardized by using potentiometry with potassium hydrogen phthalate (Sigma-Aldrich). All of the samples were prepared with freshly double distilled water. The ionic strength (*I*) was adjusted to 0.1 M by the addition of NaClO_4_ (Sigma-Aldrich).

### Potentiometric Measurements

The stability constants for proton Cu­(II) and Zn­(II) complexes with two ligands were calculated from titration curves carried out over the pH range of 2–11 at 298 K and ionic strength 0.1 M NaClO_4_. The total volume of the solution used was 3.0 mL. The potentiometric titrations were performed using a Dosimat 800 Metrohm Titrator connected to a Methrom 905 pH-meter and a Mettler Toledo pH inLab Science electrode. The thermostabilized glass cell was equipped with a magnetic stirring system, a microburet delivery tube and an inlet–outlet tube for argon. Solutions were titrated with 0.1 M carbonate-free NaOH. The electrodes were calibrated daily for hydrogen ion concentration through titrating HClO_4_ with NaOH using a total volume of 3.0 mL. The ligand concentration was 0.4 mM, and the metal-to-ligand ratios were 0.9:1. The exact concentrations and the purities of the ligand solutions were determined by the Gran method.^15^ The standard potential and the slope of the electrode couple were computed by means of GLEE program.^16^ The HYPERQUAD 2008 program was used for the stability constant calculations.^17^ The speciation diagrams were computed with the HYSS program.^18^ Hydrolysis constants for metal ions were taken from the literature.^19^ All spectroscopic experiments were performed under slightly substoichiometric conditions (M/L = 0.9:1). This approach is commonly used in Cu­(II)-peptide studies to minimize contributions from unbound Cu­(II) and to limit complications arising from Cu­(II) hydrolysis at near-neutral pH. Under these conditions, the observed spectroscopic features primarily reflect the coordination properties of the dominant mononuclear Cu­(II)-peptide complex.

### Mass Spectrometry

High-resolution mass spectra were obtained on a Bruker compact QTOF (Bruker Daltonik, Bremen, Germany), equipped with electrospray ionization source with an ion funnel. The mass spectrometer was operated in the positive ion mode. The instrumental parameters were as follows: scan range *m*/*z* 100–2000, dry gas—nitrogen, temperature 453 K, and ion energy 5 eV. The capillary voltage was optimized to the highest S/N ratio and it was 4800 V. The samples were prepared in 1:1 MeOH/H_2_O mixture at pH 7.4 with a M/L molar ratio 0.9:1, where [ligand]_tot_ = 0.1 mM. The samples were infused at a flow rate of 3 μL min^–1^. The instrument was calibrated externally with a Tunemix mixture (Bruker Daltonik, Germany) in quadratic regression mode. Data were processed by application of the Compass DataAnalysis 4.2 (Bruker Daltonik, Germany) program. The mass accuracy for the calibration was better than 5 ppm, enabling together with the true isotopic pattern (using SigmaFit) an unambiguous confirmation of the elemental composition of the obtained complex.

### Spectroscopic Studies

The absorption spectra were recorded on a Jasco-V730 spectrophotometer, in the range 200–800 nm, using a quartz cuvette with an optical path of 1 cm. Circular dichroism spectra were recorded on a Chirascan CD spectrometer in the 200–800 nm range, using a quartz cuvette with an optical path of 1 and 0.1 cm in the visible and near-UV range. The concentration of sample solutions used for spectroscopic studies was similar to those employed in the potentiometric experiment. The metal:ligand ratio was 0.9:1. All spectroscopic measurements were recorded in the pH range 3–11. The pH of the samples was adjusted with the appropriate amounts of HClO_4_ and NaOH solutions. The ε (UV–vis) and Δε (CD) values listed in the tables correspond to the individual complex species identified in the distribution diagrams and were extracted from spectra recorded at the pH values where the respective species is present at its maximum concentration. OriginPro 2016 was used to process and visualize the obtained spectra.

Electron paramagnetic resonance (EPR) spectra were recorded in liquid nitrogen on a Bruker ELEXSYS E500 CW-EPR spectrometer at X-band frequency (frequency = 9.5 GHz, modulation amplitude = 10.00 G) and equipped with an ER 036TM NMR teslameter and an E41 FC frequency counter. Ethylene glycol (25%) was used as a cryoprotectant. Ligand concentration was 1 mM and Cu­(II) to ligand ratio = 1:1. The EPR parameters were analyzed by simulating the experimental spectra using WIN-EPR SIMFONIA software, version 1.2 (Bruker).

### NMR

NMR measurements were conducted on a Bruker Avance 600 MHz spectrometer at temperatures of 288 and 298 K, using sodium 3-(trimethylsilyl)-[2,2,3,3-d4]­propanesulfonate as the internal reference for all measurements. Peptides (0.5 mM) were solubilized in 20 mM phosphate buffer (pH 7.4) containing 10% D_2_O. Zinc and Copper­(II) were added using ZnCl_2_ and Cu­(SO_4_)_2_ stocks, respectively. Spectra were processed with TopSpin 3.6. The residual water signal was suppressed using excitation sculpting (2 ms selective pulse).^20^ Proton assignments were derived from 2D TOCSY and NOESY spectra.

## Results and Discussion

A comprehensive suite of analytical techniques, including electrospray ionization mass spectrometry (ESI-MS), potentiometric titrations, UV–visible absorption spectroscopy (UV–vis), circular dichroism (CD), electron paramagnetic resonance (EPR), and nuclear magnetic resonance (NMR) spectroscopy, was employed to unravel the structural and thermodynamic properties of two domains of the CopI chaperone: the internal His/Met-rich metal-binding region (Ac-KAHAQEMRAMPDMQHAD-NH_2_) and the N-terminal His-rich fragment (Ac-DAGHDHGSAHAGAHAHDA-NH_2_).

### The internal His/Met-Rich Region of CopI (Ac-KAHAQEMRAMPDMQHAD-NH_2_)

#### MS-Based Analysis of the Stoichiometry of Cu­(II) Coordination to the Internal His/Met-Rich Region of CopI (Ac-KAHAQEMRAMPDMQHAD-NH_2_)

Electrospray ionization mass spectrometry (ESI-MS) revealed the stoichiometry of the Cu­(II) complexes with the internal His/Met-rich region of CopI Ac-KAHAQEMRAMPDMQHAD-NH_2_. Supporting Table S1 lists the *m*/*z* values for the free ligand and its metal complexes. The most intense signals (*m*/*z* = 1004.91, *z* = 2+; *m*/*z* = 670.27, *z* = 3+; *m*/*z* = 502.96, *z* = 4+) corresponds to the free peptides. Only monomeric species were detected, indicating that peptide forms 1:1 metal to ligand (M/L) complexes. The monomeric complexes [CuL]^2+^, [CuL]^3+^, [CuL]^4+^ were detected at *m*/*z* = 1034.88, 690.25 and 517.94, respectively (Figure S1 and Table S1). No evidence of polynuclear or bis-complexes was found in either ESI-MS or potentiometric measurements, further supporting a 1:1 stoichiometry under the experimental conditions.

#### Copper­(II) Interaction with the Internal His/Met-Rich Region of CopI (Ac-KAHAQEMRAMPDMQHAD-NH_2_)

For the Ac-KAHAQEMRAMPDMQHAD-NH_2_ peptide, potentiometric titrations reveal six protonation steps that can be clearly assigned to the side-chain donors (Table 1). The highest constant (log*K* = 9.75) corresponds to the ε-NH_2_ group of Lys, followed by two successive protonation steps with log*K* = 7.01 and 6.18, which are attributed to the N_im_ atoms of the two His residues. At lower pH, the carboxylate groups are protonated in the order Glu (log*K* = 4.30) and then the two Asp residues (log*K* = 3.73 and 2.67).^21–23^

**Table 1:** Protonation constants for Ac-KAHAQEMRAMPDMQHAD-NH_2_ ligand at *T* = 298 K and *I* = 0.1 M (NaClO_4_)­^a^

species	logβ	log*K*	aa
HL	9.75(1)	9.75	–εNH_2_ (Lys)
H_2_L	16.76(2)	7.01	–N_im_ (His)
H_3_L	22.94(2)	6.18	–N_im_ (His)
H_4_L	27.24(3)	4.30	–O_COO_^–^ (Glu)
H_5_L	30.97(3)	3.73	–O_COO_^–^ (Asp)
H_6_L	33.64(5)	2.67	–O_COO_^–^ (Asp)

a Values in parentheses are standard deviations on the last significant figure.

In the case of the Cu­(II)-Ac-KAHAQEMRAMPDMQHAD-NH_2_ complex, five mononuclear species are present in the studied pH range (Table 2 and Figure 1). The first form, CuH_2_L, dominates at pH 5 and corresponds to initial Cu­(II) coordination by one His imidazole nitrogen. The d-d band at 686 nm with a low molar absorptivity (ε = 77 M^–1^ cm^–1^) (Figure S2) and EPR parameters *A*_II_ = 157 G and *g*_II_ = 2.32 (Figure S4) clearly indicate a 1N_im_ binding mode.^24,25^ The CD spectra exhibit a characteristic ligand-to-metal charge-transfer (LMCT) band associated with N_**im**_ → Cu­(II) excitation, typically observed in the 250–300 nm range (Figure S3). The next species, CuHL, becomes predominant near pH 6–7 and is formed by deprotonation of the second His residue (Table 2 and Figure 1). The appearance of the d-d transition at 630 nm with ε = 126 M^–1^ cm^–1^ (Figure S2), together with EPR values *A*_II_ = 164 G and *g*_II_ = 2.27 (Figure S4), confirms the involvement of two imidazole nitrogens 2N_im_ in Cu­(II) coordination.^26^ This assignment is consistent with the p*K*_a_ value of 5.40, lowered compared to the free ligand, which indicates metal-assisted deprotonation of His (Tables 1, and 2). The next species CuH_–1_L which reaches its maximum concentration around pH 8, results from the deprotonation and subsequent coordination of two amide nitrogens (Table 2 and Figure 1). These deprotonation steps occur within a very narrow pH range; therefore, the intermediate CuL species is not resolved in the speciation model. The presence of a characteristic CD band at 332 nm (Δε = −0.47 M^–1^ cm^–1^) clearly indicates the involvement of amide nitrogens in the coordination sphere (Figure S3). Simultaneously, the UV–vis transition at 588 nm (ε = 167 M^–1^ cm^–1^) supports a 3N donor set (Figure S2), in which one of the histidine donors is replaced by a deprotonated amide, giving rise to a 1N_im_, 2N^–^ binding mode.^27–29^ Further deprotonation leads to the formation of the CuH_–2_L species, with maximum concentration at pH 9.5 (Table 2 and Figure 1). At this stage, the UV–vis spectrum shows a shift of the d-d band to 522 nm and an increase in ε to 196 M^–1^ cm^–1^, characteristic of the next amide nitrogen coordination (Figure S2). Together with EPR values *A*_II_ = 180 G and *g*_II_ = 2.20 (Figure S4), these spectroscopic features confirm the formation of a strong-field 4N donor set 1N_im_, 3N^–^ resulting from stepwise amide deprotonation.^30,31^ The CD spectrum, with a negative band near 496 nm and a positive Cotton effect around 642 nm, provides additional evidence for square-planar geometry typical of Cu­(II) complexes with an 4N chromophore (Figure S3). The last observed species, CuH_–3_L, predominates above pH 10.5 (Table 2 and Figure 1). The further hypsochromic shift of the d-d band to 508 nm (ε = 277 M^–1^ cm^–1^) (Figure S2), combined with EPR parameters *A*_II_ = 185 G and *g*_II_ = 2.19 (Figure S4), is consistent with the formation of a highly nitrogen-coordinated square-planar Cu­(II) complex involving four amide nitrogens in the coordination sphere. An alternative interpretation is the retention of the same binding mode as in the CuH_–2_L species, accompanied by the deprotonation of noncoordinating lysine residues (the p*K*_a_ value for this deprotonation in the Cu­(II) complex, 9.86, is essentially identical to that of the free ligand, 9.75) (Table 2).^23,32^

**Figure 1:**
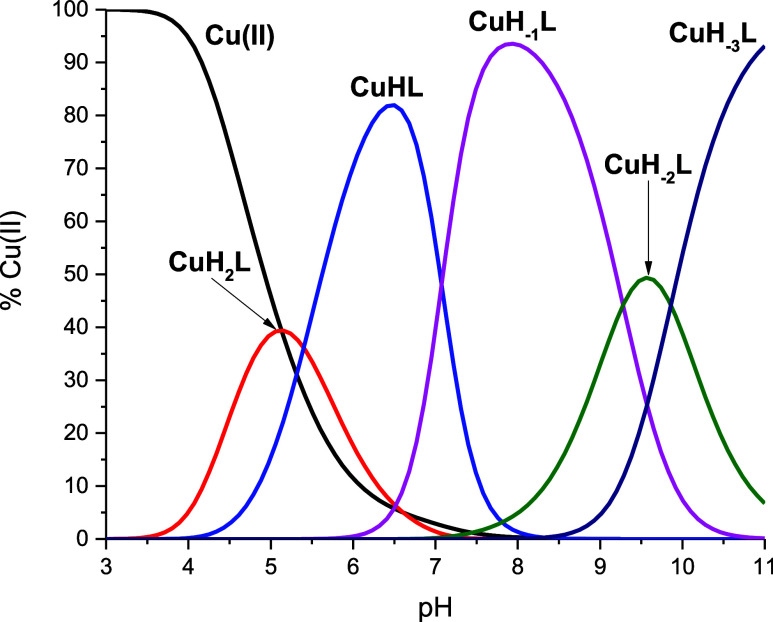
Distribution diagrams for the formation of Cu­(II) complex with Ac-KAHAQEMRAMPDMQHAD-NH_2_, *T* = 298 K and *I* = 0.1 M (NaClO_4_), M/L molar ratio = 0.9:1, *C*_L_ = 0.4 mM.

**Table 2:** Equilibrium constants, spectroscopic parameters and proposed coordination modes for Cu­(II)-Ac-KAHAQEMRAMPDMQHAD-NH_2_ at *T* = 298 K and *I* = 0.1 M (NaClO_4_)­^a^

			UV–vis		CD		EPR	
species and proposed coordination mode	logβ*_ijk_* ^b^	p*K*_a_ ^c^	λ [nm]	ε [M^–1^ cm^–1^]	λ [nm]	Δε [M^–1^ cm^–1^]	*A*_II_	*g*_II_
CuH_2_L 1N_im_	21.31(5)		686	77.21	260	0.12	157	2.32
CuHL 2N_im_	15.91(4)	5.40	630	126.32	255	0.54	164	2.27
CuH_–1_L 1N_im_, 2N^–^	1.76(5)		588	167.44	252	4.34	170	2.22
					332	–0.47		
					530	0.27		
CuH_–2_L 1N_im_, 3N^–^	–7.52(8)	9.28	522	195.80	255	4.33	180	2.20
					312	0.47		
					350	–0.16		
					496	–0.42		
					642	0.38		
CuH_–3_L 1N_im_, 3N^–^/4N^–^	–17.38(8)	9.86	508	276.55	259	4.14	185	2.19
					313	0.81		
					356	–0.22		
					497	–0.76		
					641	0.72		

a M/L ratio was 1:1. Values in parentheses are standard deviations on the last significant figure.

b Cu­(II) stability constants are presented as cumulative logβ*_ijk_* values. L stands for a fully deprotonated peptide ligand that binds Cu­(II) ions: β­(M*_i_* H*_j_* L*_k_*) = [M*_i_* H*_j_* L*_k_*]/([M]*^i^* [H]*^j^* [L]*^k^*), where [L] is the concentration of the fully deprotonated peptide.

c p*K*_a_ values of the peptides were derived from cumulative constants: p*K*_a_ = logβ­(H*_j_* L*_k_*) – logβ­(H_*j*–1_L*_k_*). For Cu­(II) complexes: p*K*_a_ = logβ­(M*_i_* H*_j_* + 1L*_k_*) – logβ­(M*_i_* H*_j_* L*_k_*).

To complement the potentiometric and spectroscopic data and to obtain residue-specific information on Cu­(II) binding, NMR spectroscopy was employed. The experiments were performed at pH 7.4, corresponding to near-physiological conditions. The NMR results provide insights into the involvement of individual residues within the His/Met-rich region of CopI in copper coordination. As evident from the superimposition of the 1D spectra of Ac-KAHAQEMRAMPDMQHAD-NH_2_ recorded in absence and in the presence of 0.05 Cu­(II) eqs (Figure 2), the substoichiometric addition of Cu­(II) to the peptide induces a severe broadening of the signals corresponding to the imidazole protons of His3 and His15. This effect is a hallmark of their close proximity to the paramagnetic center. Interestingly, the spectra also exhibit a milder broadening of the Gln side-chain amide resonances (δNH_2_). This observation supports the direct participation of both His in the coordination sphere, as the paramagnetic effect propagates to the adjacent Gln5 and Gln14 residues.

**Figure 2:**
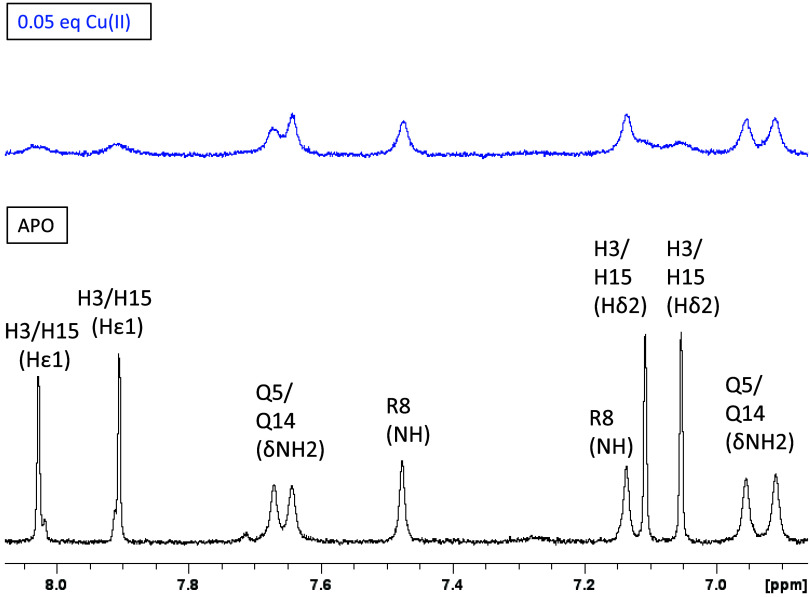
Superimposition of the 1D spectra of Ac-KAHAQEMRAMPDMQHAD-NH_2_ recorded in absence and in the presence of 0.05 Cu­(II) eqs *T* = 288 K, *C*_M_ = 0.5 mM, phosphate buffer = 20 mM, pH 7.4.

Similarly, the presence of Cu­(II) induces selective line broadening on the scalar cross-peaks of 2D ^1^H–^1^H TOCSY NMR spectra, leading to significant reduction of their intensities (Figure 3). In the amide region (Figure 3A), the NH-Hβ correlations of the histidine residues (H3/H15) undergo severe broadening, rendering them nearly undetectable. A similar effect is observed for the NH-Hβ cross-peak of one of the aspartic acid residues (D12 or D17), while the other Asp signal remains less affected. Regarding the NH-Hγ correlations of the methionines (M7/M10/M13), all signals exhibit intensity reduction; however, one specific resonance undergoes a much more pronounced broadening compared to the other two, appearing almost completely suppressed. Furthermore, the overlapping signals corresponding to the NH-Hγ correlations of the glutamine and glutamate residues (Q5/E6/Q14) are completely broadened out, resulting in the total disappearance of these cross-peaks. Consistently, the aliphatic region (Figure 3B) exhibits marked broadening of the Hα-Hβ correlations. This effect is particularly intense for the H3/H15 spin systems. Moreover, the cross-peaks for both aspartic acid residues (D12 and D17) are completely absent in the presence of the metal, strongly suggesting the involvement of the carboxylate groups in metal binding. Finally, the paramagnetic influence is also observable on the N-terminal residue, affecting the Hα-Hε correlation of Lys1.

**Figure 3:**
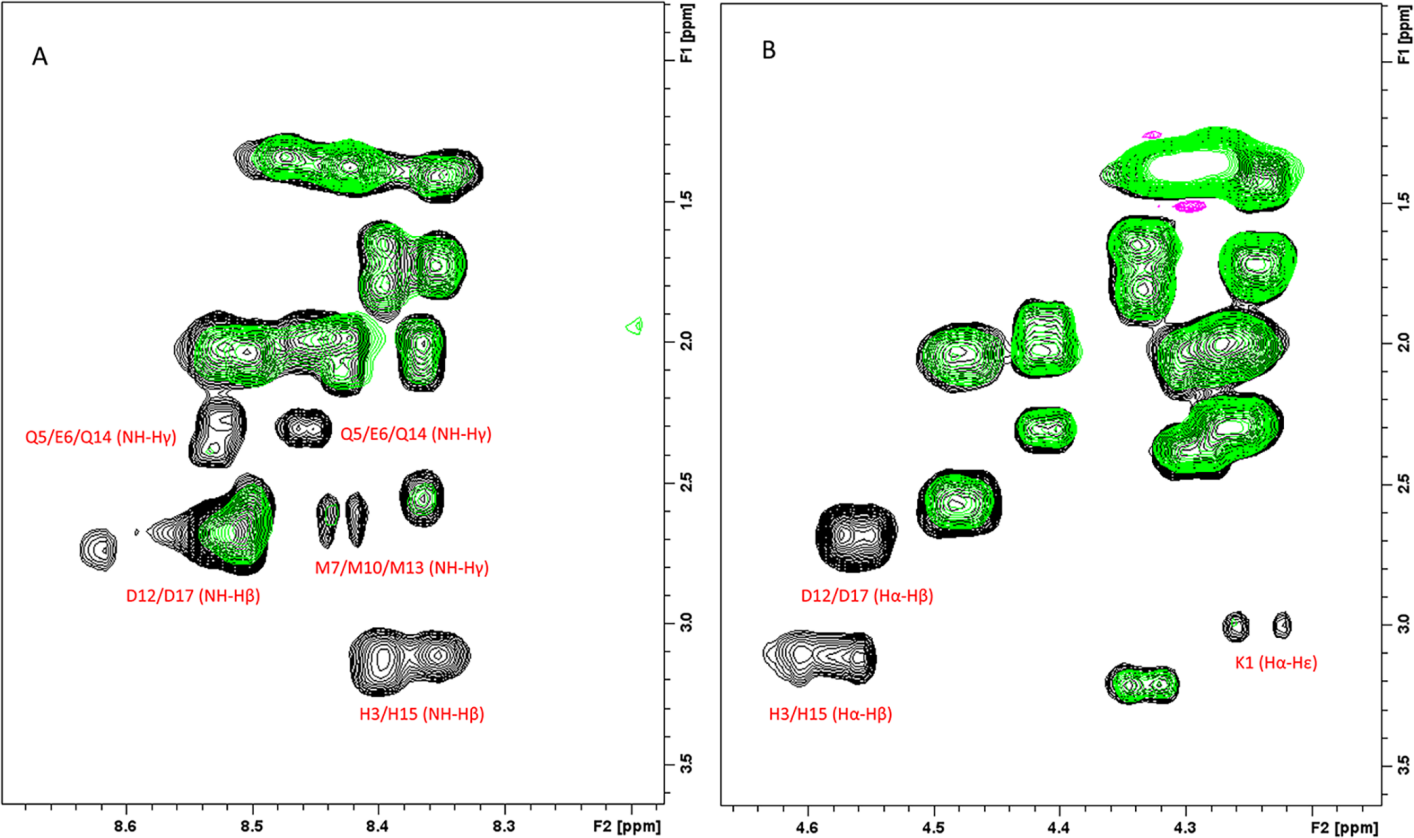
Superimposition of the (A) fingerprint and (B) aliphatic regions of 2D ^1^H–^1^H TOCSY spectra of Ac-KAHAQEMRAMPDMQHAD-NH_2_ recorded in absence (black) and in the presence of 0.05 Cu­(II) eqs (green). *T* = 288 K, *C*_M_ = 0.5 mM, phosphate buffer = 20 mM, pH 7.4_._.

In conclusion, the NMR data, together with previously reported results, indicate that at pH 7.4 Cu­(II) simultaneously coordinates the imidazole rings of His3 and His15. This coordination is consistent with a metal-induced peptide folding event that brings these two residues into close spatial proximity, accompanied by the involvement of the carboxylate groups of Asp12 and Asp17. Such a conformational rearrangement is supported by the pronounced line broadening observed for the intervening residues (Met, Gln, Asp) as well as for Lys1, consistent with their location within the immediate paramagnetic environment generated by the coordinated Cu­(II) ion. Furthermore, the complete disappearance of one methionine signal in the NMR spectrum upon addition of Cu­(II) may suggest the direct involvement of a methionine residue in metal binding. Notably, a similar behavior has been reported for Cu­(I) coordination to the internal domain of CopI, where methionine residues are proposed to serve as donor ligands.^8^ Taken together, these observations support a coordination mode involving two histidine residues, one methionine sulfur donor, and an aspartate carboxylate group (Figure 4).

**Figure 4:**
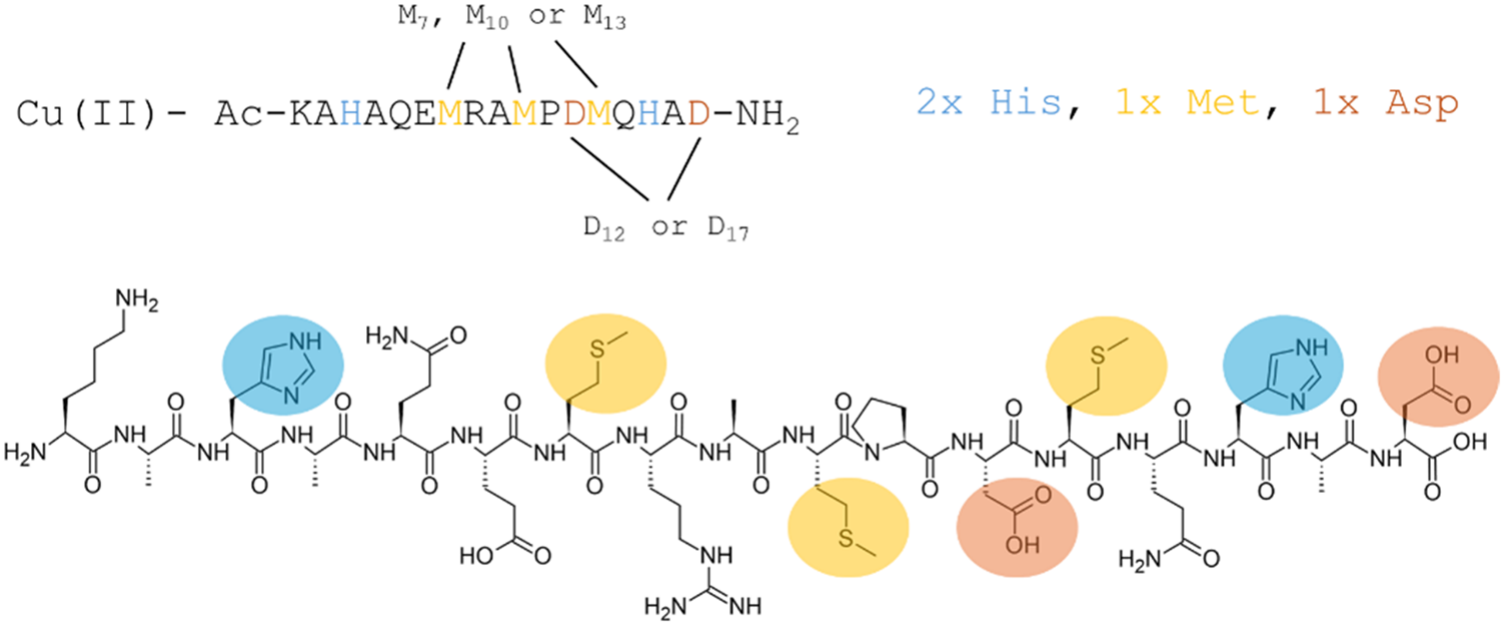
Proposed Cu­(II) coordination mode in Ac-KAHAQEMRAMPDMQHAD-NH_2_ at pH 7.4 based on potentiometric, spectroscopic and NMR data.

#### Structural Properties of the His/Met-Rich Region of CopI and Its Cu­(II) Complexes

Circular dichroism spectroscopy was employed to evaluate the secondary-structure preferences of the His/Met-rich region of CopI across a broad pH range and to assess the structural impact of Cu­(II) coordination (Figure 5). In the apo form, the peptide exhibits CD spectra characteristic of a predominantly disordered ensemble across the entire pH range. All spectra display a strong negative band at 197–200 nm, typical of intrinsically disordered peptides. While the intensity of this minimum shows modest pH dependence, its overall shape remains largely unchanged, indicating the absence of significant stabilization of regular secondary structure under any of the tested conditions. Only minor pH-dependent variations in elipticity are observed, which can be attributed to changes in protonation of His residues rather than to any stabilization of defined structural motifs (Figure 5A). Upon Cu­(II) binding, the CD profiles exhibit small but clearly detectable changes, particularly at pH values above 7. The weak negative band around 230 nm disappears, while a broad positive feature emerges in the 240–260 nm region. These spectral perturbations are consistent with a shift in the coordination environment of Cu­(II), reflecting increased involvement of deprotonated histidine residues and, at higher pH, the recruitment of deprotonated amide donors (Figure 5B).

**Figure 5:**
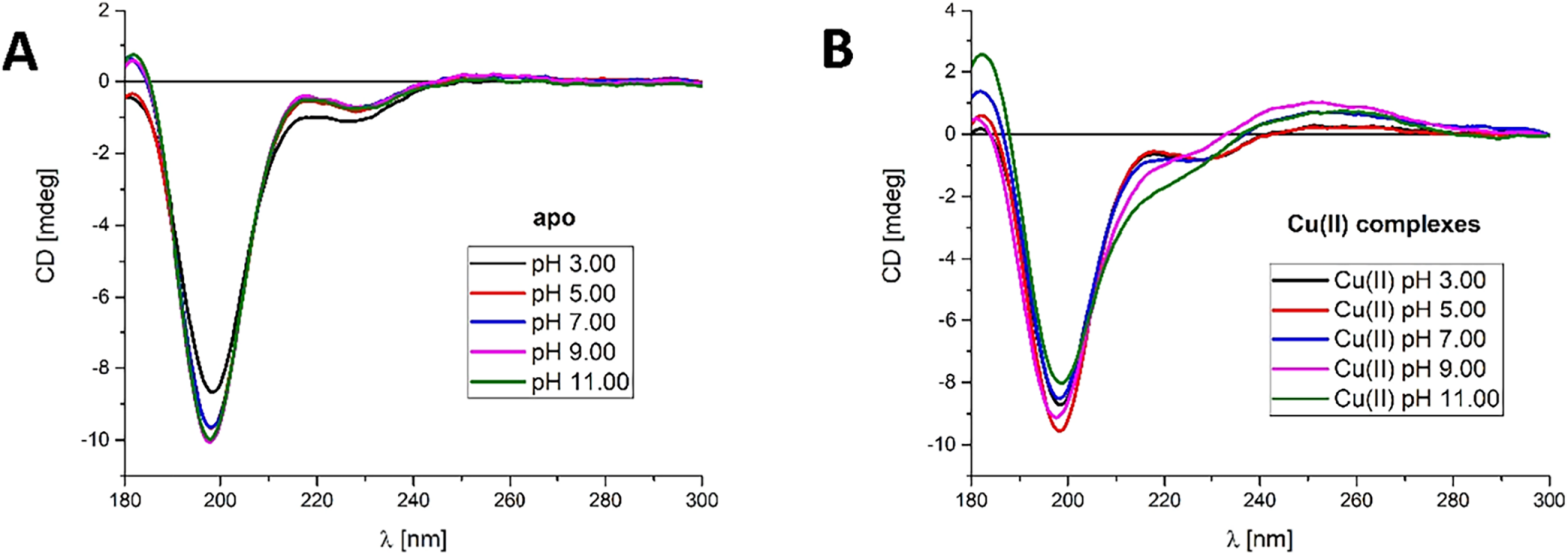
CD spectra for (A) Ac-KAHAQEMRAMPDMQHAD-NH_2_ (B) Cu­(II)-Ac-KAHAQEMRAMPDMQHAD-NH_2_ in aqueous solution of 4 mM HClO_4_ with *I* = 0.1 M NaClO_4_, *T* = 298 K, M/L molar ratio = 0.9:1, *C*_L_ = 0.1 mM.

### The N-Terminal His-Rich Domain of CopI (Ac-DAGHDHGSAHAGAHAHDA-NH_2_)

#### Stoichiometry of Cu­(II) Binding to the N-Terminal His-Rich Domain of CopI (Ac-DAGHDHGSAHAGAHAHDA-NH_2_) Revealed by ESI-MS

Electrospray ionization mass spectrometry (ESI-MS) was employed to characterize the Cu­(II) binding properties of the N-terminal His-rich fragment of CopI, Ac-DAGHDHGSAHAGAHAHDA-NH_2_. The experimental spectra revealed a set of well-defined signals corresponding to the free peptide and its copper complexes (Table S2 and Figure S5). The dominant peaks assigned to the free ligand were observed at *m*/*z* = 887.89 (*z* = 2+) and *m*/*z* = 592.27 (*z* = 3+), confirming the monomeric nature of the peptide under the applied conditions. For Cu­(II) complexes the species [CuL]^2+^ and [CuL]^3+^ were detected at *m*/*z* = 918.34, and 612.58, respectively, and their isotopic patterns were fully consistent with the presence of a single Cu­(II) ion bound to one peptide molecule. Additional sodium-containing adducts were also detected for both the free ligand and its Cu­(II) complexes. For the peptide, ions such as [L + Na]^2+^ (*m*/*z* = 898.88), [L + 2Na]^2+^ (*m*/*z* = 909.86) and their corresponding 3+ charged forms, including [L + Na]^3+^ (*m*/*z* = 599.81) and [L + 2Na]^3+^ (*m*/*z* = 606.92), were observed. Analogous sodium adducts were found for the Cu­(II) complex [CuL + Na]^2+^ (*m*/*z* = 929.33), [CuL + 2Na]^2+^ (*m*/*z* = 940.32) as well as [CuL + Na]^3+^ (*m*/*z* = 619.90) and [CuL + 2Na]^3+^ (*m*/*z* = 627.23).

#### Copper­(II) Interaction with the N-Terminal His-Rich Domain of CopI (Ac-DAGHDHGSAHAGAHAHDA-NH_2_)

Potentiometric titrations of the Ac-DAGHDHGSAHAGAHAHDA-NH_2_ peptide reveal eight sequential protonation steps (Table 3). The first five protonation constants (log*K* = 7.51, 6.97, 6.50, 6.23, 5.47) correspond to the stepwise protonation of the imidazole nitrogen atoms of the histidine residues. The remaining protonation steps, with log*K* values of 3.79, 3.52, and 2.16, are assigned to the protonation of the Asp carboxylate groups (O_COO_^–^). The p*K* values obtained from the potentiometric data are in good agreement with those found in the literature for similar poly-His systems.^22,33–35^

**Table 3:** Protonation constants for Ac-DAGHDHGSAHAGAHAHDA-NH_2_ ligand at *T* = 298 K and *I* = 0.1 M (NaClO_4_)­^a^

species	logβ	log*K*	aa
HL	7.51(1)	7.51	–N_im_ (His)
H_2_L	14.48(1)	6.97	–N_im_ (His)
H_3_L	20.98(2)	6.50	–N_im_ (His)
H_4_L	27.21(1)	6.23	–N_im_ (His)
H_5_L	32.68(2)	5.47	–N_im_ (His)
H_6_L	36.47(3)	3.79	–O_COO_^–^ (Asp)
H_7_L	39.99(3)	3.52	–O_COO_^–^ (Asp)
H_8_L	42.15(5)	2.16	–O_COO_^–^ (Asp)

a Values in parentheses are standard deviations on the last significant figure.

According to potentiometric data calculations, Cu­(II) forms seven species with Ac-DAGHDHGSAHAGAHAHDA-NH_2_ CopI N-terminal His-rich domain characterized by the logβ and p*K*_a_ values collected in Table 4. The distribution diagram for the obtained Cu­(II) complexes is shown in Figure 6. The first complex detected at low pH is CuH_4_L with a maximum concentration at pH 4.2 (Table 4 and Figure 6). It is most probable that in this complex one imidazole residue is coordinated to Cu­(II), although binding to the carboxylate group can also be possible. The next species, CuH_3_L (pH 4.6) indicates the formation of a 2N_im_ complex, which is supported by the presence of a d**-**d band at 648 nm (ε = 70 M^–1^ cm^–1^), suggesting coordination of a second imidazole nitrogen (Figure S6). The EPR parameters (*A*_II_ = 173 G and *g*_II_ = 2.27) also confirm a 2N coordination mode (Figure S8). For the following species, CuH_2_L, the amide nitrogen begins to participate in the Cu­(II) coordination sphere. The CD spectrum clearly shows a band at 323 nm (Δε = −0.26 M^–1^ cm^–1^) that appears above pH 5, corresponding to an amide-Cu­(II) charge-transfer transition (Figure S7). Additionally, a positive band at 552 nm (Δε = 0.29 M^–1^ cm^–1^) indicates a “not fully” planar square geometry, consistent with a 3N binding mode involving two imidazole nitrogens and one deprotonated amide nitrogen 2N_im_, N^–^ (Figure S7). The blue shift of the UV–vis band to 578 nm also supports the presence of a 3N complex (Figure S6). The next two species, CuHL and CuL, exhibit the same binding mode, resulting from deprotonation of noncoordinating histidine residues. Their UV–vis and CD spectra closely resemble those of CuH_2_L, confirming that the coordination mode remains unchanged. The EPR parameters likewise indicate a 3N environment (*A*_II_ = 182 G and *g*_II_ = 2.24 and *A*_II_ = 186 G and *g*_II_ = 2.24, respectively). Notably, the p*K*_a_ values of these complexes (5.53 and 6.50) (Table 4) are lower than those of the apo form (6.50 and 6.97) (Table 3), suggesting dynamic exchange among all imidazole residues in the sequence, even though only two histidines can coordinate simultaneously. This behavior has been previously observed.^33,35^ The next species, CuH_–2_L, arises from subsequent deprotonation of additional amide nitrogen, forming 2N_im_, 2N^–^ complex (Table 4 and Figure 6). The last species, CuH_–3_L, is associated with the deprotonation of a polymorphic states, as reported previously third amide nitrogen (p*K*_a_ = 9.52, 1N_im_, 3N^–^). A 4N coordination mode for the last two species is supported by the blue-shifted UV–vis bands observed for these species (556 and 546 nm) (Figure S6). The presence of square-planar complexes is further confirmed by positive Cotton effects at 618 and 651 nm and negative Cotton effects at 544 and 564 nm in the CD spectra (Figure S7). These findings are consistent with the EPR parameters, which also indicate 4N coordination (*A*_II_ = 191 G and *g*_II_ = 2.20 and *A*_II_ = 198 G and *g*_II_ = 2.19, respectively) (Figure S8).^26,36^

**Figure 6:**
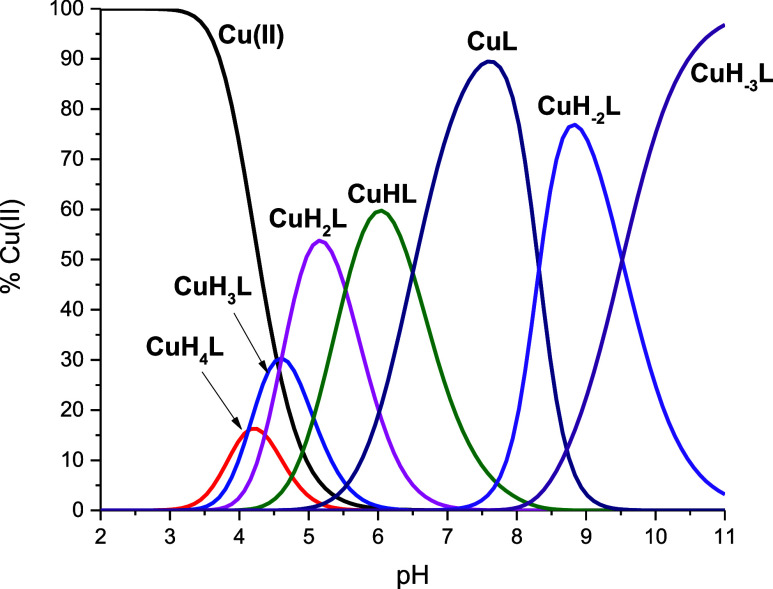
Distribution diagrams for the formation of Cu­(II) complex with Ac-DAGHDHGSAHAGAHAHDA-NH_2_, *T* = 298 K and *I* = 0.1 M (NaClO_4_), M/L molar ratio = 0.9:1, *C*_L_ = 0.4 mM.

**Table 4:** Equilibrium constants, spectroscopic parameters and proposed coordination modes for Ac-DAGHDHGSAHAGAHAHDA-NH_2_ at *T* = 298 K and *I* = 0.1 M (NaClO_4_)­^a^

			UV–vis		CD		EPR	
species and proposed coordination mode	logβ*_ijk_* ^b^	p*K*_a_ ^c^	λ [nm]	ε [M^–1^ cm^–1^]	λ [nm]	Δε [M^–1^ cm^–1^]	*A*_II_	*g*_II_
CuH_4_L 1N_im_	31.37(6)							
CuH_3_L 2N_im_	27.24(2)	4.13	648	69.58			173	2.27
CuH_2_L 2N_im_, 1N^–^	22.63(2)	4.61	578	143.05	552	0.29		
					323	–0.26		
CuHL 2N_im_, 1N^–^	17.10(1)	5.53	578	82.15	548	0.43	182	2.24
					317	–0.35		
CuL 2N_im_, 1N^–^	10.60(2)	6.50	578	84.82	545	0.49	186	2.24
					321	–0.40		
CuH_–2_L 2N_im_, 2N^–^	–6.04(3)		556	83.68	618	0.36	191	2.20
					544	–0.08		
					488	0.13		
					334	–0.70		
					255	3.05		
CuH_–3_L 1N_im_, 3N^–^	–15.56(4)	9.52	546	144.69	651	0.13	198	2.19
					564	–0.36		
					497	0.12		
					340	–0.18		
					265	2.12		

a M/L ratio was 1:1. Values in parentheses are standard deviations on the last significant figure.

b Cu­(II) stability constants are presented as cumulative logβ*_ijk_* values. L stands for a fully deprotonated peptide ligand that binds Cu­(II) ions: β­(M*_i_* H*_j_* L*_k_*) = [M*_i_* H*_j_* L*_k_*]/([M]*^i^* [H]*^j^* [L]*^k^*), where [L] is the concentration of the fully deprotonated peptide.

c p*K*_a_ values of the peptides were derived from cumulative constants: p*K*_a_ = log β­(H*_j_* L*_k_*) – logβ­(H_*j*–1_L*_k_*). For Cu­(II) complexes: p*K*_a_ = logβ­(M*_i_* H*_j_* + 1L*_k_*) – logβ­(M*_i_* H*_j_* L*_k_*).

Spectroscopic studies on the full-length CopI protein^14^ suggested that the N-terminal copper-binding site may involve histidine residues together with the free N-terminal amine, giving rise to 3N1O coordination environment. In particular, participation of two histidine imidazole donors together with the N-terminal amino group was proposed. In our work, the N-terminal His-rich region was investigated using a synthetic peptide with blocked termini. This design intentionally eliminates the possibility of coordination through the free N-terminal amine and therefore allows the intrinsic coordination properties of the histidine-rich sequence itself to be evaluated more directly. Under these conditions, the spectroscopic and potentiometric data consistently indicate that no more than two histidine imidazole nitrogens participate in Cu­(II) coordination. With increasing pH, the remaining coordination positions are progressively occupied by deprotonated backbone amide nitrogens, which is consistent with the well-established coordination behavior of Cu­(II) in histidine-containing peptides. Importantly, our data do not support the formation of a coordination mode involving three histidine imidazole donors within this sequence fragment. Thus, while previous studies suggested the involvement of histidine residues in the N-terminal Cu­(II) site of CopI, the present peptide model indicates that the intrinsic coordination chemistry of this region favors a maximum of two imidazole donors, with additional stabilization provided by backbone amide coordination at higher pH.

NMR spectroscopy was next applied to investigate the Cu­(II) interaction with the N-terminal His-rich CopI domain at pH 7.4. The addition of Cu­(II) induces a complex pattern of selective line broadening in the TOCSY spectra of the Ac-DAGHDHGSAHAGAHAHDA-NH_2_ peptide (Figure 7). Due to significant signal overlap, unambiguous assignment of individual residues within the same amino acid type is often precluded; however, distinct paramagnetic effects are clearly observable. In the region corresponding to the alanine residues (A2/A9/A11/A13/A15/A18) the overlay reveals that approximately three out of the six alanine NH-Hβ correlations undergo marked broadening (Figure 7A), while the remaining signals appear relatively unaffected. Regarding the aspartic acid residues (D1/D5/D17), the NH-Hβ region shows that one specific resonance is significantly more broadened compared to the other two, which retain higher signal intensity (Figure 7B). The most pronounced and widespread effects involve the five histidine residues. In the amide region (Figure 7B), the NH-Hβ correlation of one histidine is completely broadened out, resulting in the total loss of the signal. The corresponding cross-peaks for the other four histidines are also strongly affected, exhibiting severe line broadening. This intense paramagnetic influence is further confirmed in the aromatic side-chain region (Figure 7C): the correlations between the Hδ2 and Hβ protons display a mix of total signal disappearance for some residues and strong broadening for others, although the severe overlap prevents specific attribution. Finally, a milder paramagnetic effect is observed for the serine (S8) residue, whose NH-Hβ cross-peak exhibits only a slight reduction in intensity.

**Figure 7:**
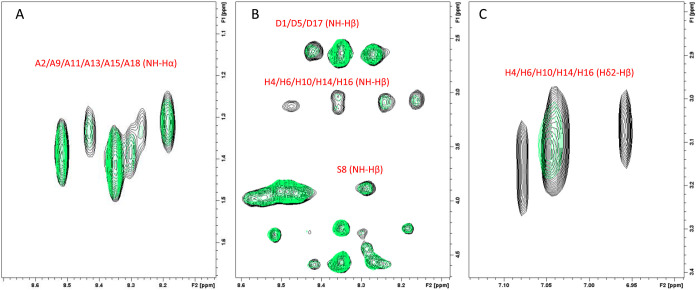
Superimposition of the (A, B) fingerprint and (C) aromatic regions of 2D ^1^H–^1^H TOCSY spectra of Ac-DAGHDHGSAHAGAHAHDA-NH_2_ recorded in absence (black) and in the presence of 0.05 Cu­(II) eqs (green). *T* = 288 K, *C*_M_ = 0.5 mM, phosphate buffer = 20 mM, pH 7.4_._.

Considering the structural data, the interaction is indicative of a multihistidine coordination mode. The widespread broadening involving all five histidine residues, strongly suggests that the copper ion may adopt polymorphic states, as reported previously.^33,35,37^ Additionally, the pattern of broadening, affecting approximately three alanines and one aspartate while sparing the central serine S8, strongly points to the formation of a folded structure involving the C-terminal histidine-rich motif including H10, H14 and H16. In this scenario, the metal ion likely anchors to the histidines in this region, trapping the intervening alanines (A11, A13 and A15) and the adjacent C-terminal aspartate (D17) within the paramagnetic sphere, while the upstream residues (around S8) remain more flexible and distant from the core.

To conclude, although previous potentiometric and spectroscopic studies established that only two histidine residues are coordinated to Cu­(II) at any given time, the NMR data reveal fast dynamic exchange among all imidazole rings. This behavior indicates the formation of polymorphic Cu­(II) complexes in which different pairs of histidine residues transiently participate in metal binding. Such dynamic polymorphism is consistent with the previously observed decrease in the p*K*_a_ values of all histidine residues upon complex formation relative to the free ligand, despite the fact that only two histidines are directly involved in Cu­(II) coordination at any given moment, as demonstrated by UV–vis and CD spectroscopy. Overall, the NMR results support the presence of a rapidly interconverting ensemble of coordination isomers, giving rise to exchange-averaged behavior on the NMR time scale. For clarity, Figure 8 highlights all histidine residues affected in the NMR spectra, together with the most probable additional donor groups inferred from spectroscopic data, including a deprotonated amide nitrogen and a water molecule completing the coordination sphere.

**Figure 8:**
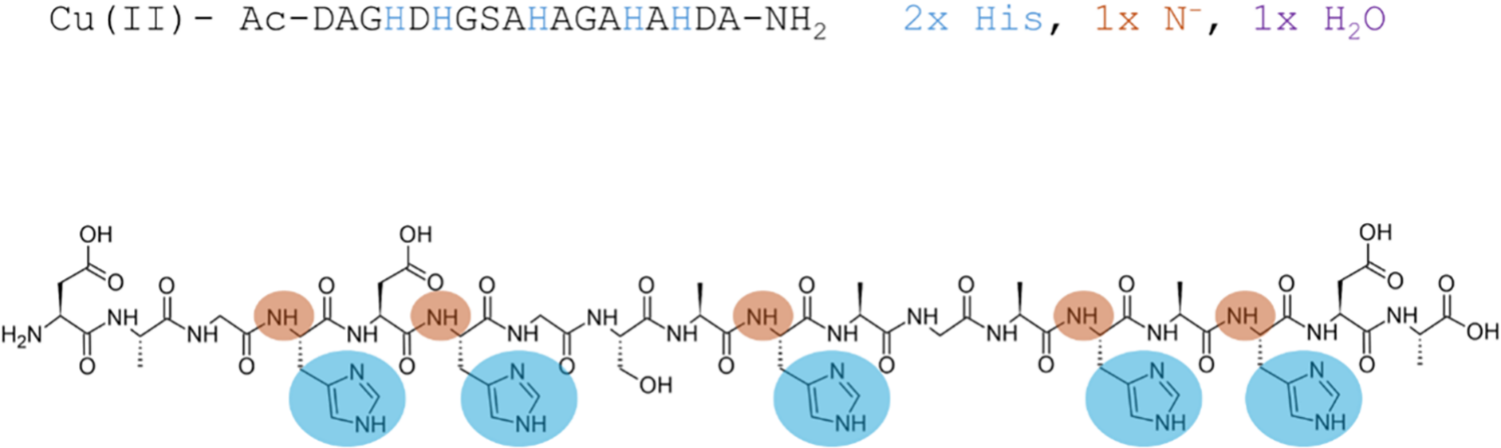
Proposed Cu­(II) coordination mode in Ac-DAGHDHGSAHAGAHAHDA-NH_2_ at pH 7.4 based on potentiometric, spectroscopic and NMR data (amide nitrogens were selected based on the well-established Cu­(II) coordination pattern, in which, upon histidine anchoring, deprotonation and coordination of the preceding (N-terminal) amide groups occur).

#### Structural Analysis of the N-Terminal His-Rich Domain and Its Cu­(II) Complexes

As observed previously for the internal His/Met-rich fragment of CopI, the N-terminal His-rich domain displays CD characteristics of a predominantly disordered peptide throughout the entire pH range. In its apo form, the spectrum is dominated by a strong minimum at 198–200 nm (Figure 9A). No evidence for the formation or stabilization of regular secondary-structure elements is detected under any of the tested conditions. Coordination of Cu­(II) induces small but clearly resolvable perturbations in the spectral profiles (Figure 9B). The most pronounced changes occur at neutral and basic pH, where the decrease of the negative contribution near 230 nm and the emergence of a broad positive band in the 240–260 nm region point to modifications in the metal-binding environment. These features are consistent with increased participation of deprotonated imidazole donors and, at higher pH, recruitment of backbone amide nitrogens into the Cu­(II) coordination sphere. Although Cu­(II) binding induces detectable spectral changes in the Ac-DAGHDHGSAHAGAHAHDA-NH_2_ peptide, no signatures of α-helical structure are observed at any pH. This behavior contrasts with previously reported Cu­(II)-His_6_ peptides,^33^ in which metal coordination promotes or stabilizes α-helical conformations. The difference likely arises from the sequence architecture: whereas His_6_ motifs contain six consecutive histidine residues capable of forming a compact, cooperatively stabilized metal-binding environment compatible with helix formation, the N-terminal His-rich domain of CopI features five histidines dispersed throughout the sequence and separated by helix-disrupting residues such as glycine and aspartate. This interrupted arrangement prevents the alignment of imidazole side chains required to form or stabilize an α-helix upon Cu­(II) coordination. As a consequence, Cu­(II)-Ac-DAGHDHGSAHAGAHAHDA-NH_2_ complexes undergo only local changes in the coordination sphere rather than adopting any defined secondary structure.

**Figure 9:**
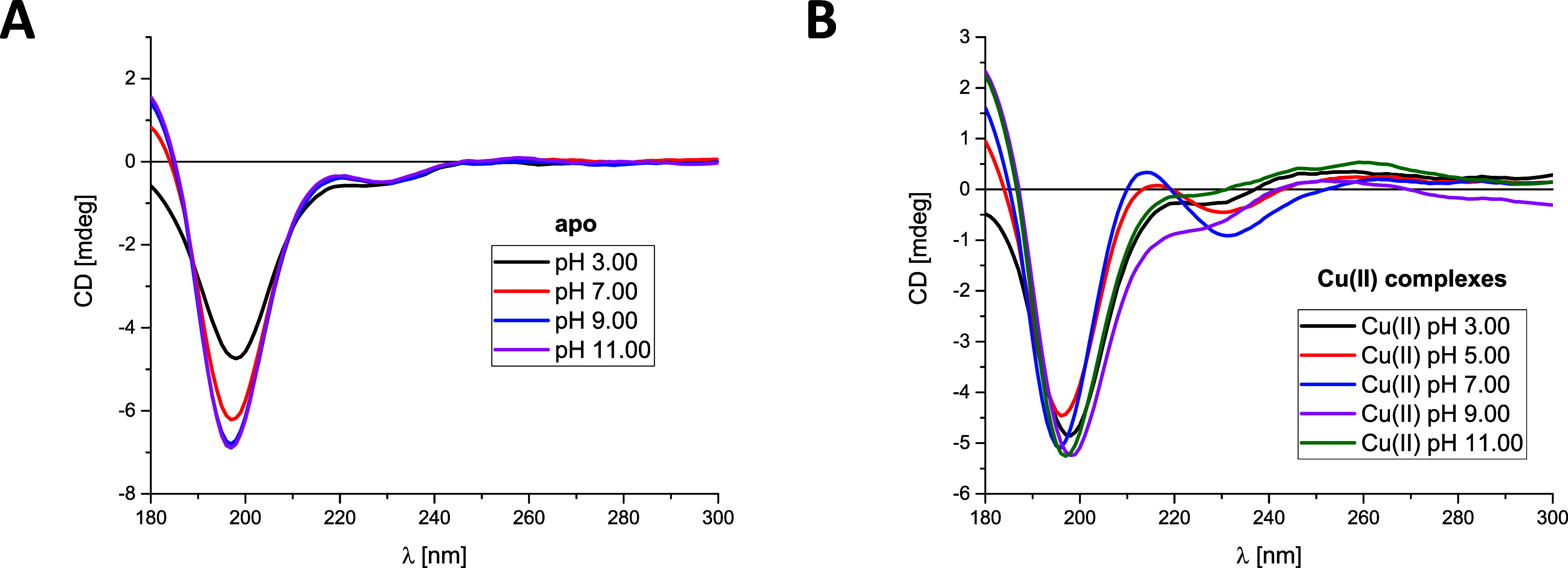
CD spectra for (A) Ac-DAGHDHGSAHAGAHAHDA-NH_2_ (B) Cu­(II)- Ac-DAGHDHGSAHAGAHAHDA-NH_2_ in aqueous solution of 4 mM HClO_4_ with *I* = 0.1 M NaClO_4_, *T* = 298 K, M/L molar ratio = 0.9:1, *C*_L_ = 0.1 mM.

#### Internal His/Met-Rich vs N-Terminal His-Rich Domain—Potentiometric Differences

To directly compare the Cu­(II)-binding abilities of the two metal interacting regions of CopI, a competitive species-distribution analysis was performed for the internal His/Met-rich domain (Ac-KAHAQEMRAMPDMQHAD-NH_2_) and the N-terminal His-rich domain (Ac-DAGHDHGSAHAGAHAHDA-NH_2_). The resulting competition plot is shown in Figure 10. The N-terminal His-rich domain exhibits dominant Cu­(II) binding across a wide pH range. Under physiologically relevant conditions (pH 6–7.4), this domain overwhelmingly outcompetes the internal His/Met-rich sequence, retaining the vast majority of Cu­(II). This indicates that although the internal domain contains both histidine and methionine residues, its effective Cu­(II) affinity is markedly lower than that of the N-terminal sequence. Such behavior is consistent with earlier observations for histidine-rich peptides, where a higher density of histidine residues directly correlates with increased complex stability and tighter Cu­(II) binding.^35,38,39^ Importantly, these findings are also in agreement with the results reported by Rossotti et al.,^8^ who demonstrated that in the full-length CopI protein the N-terminal region constitutes the primary Cu­(II)-binding site, while the second site displays lower affinity. Therefore, our results obtained for peptide models mimicking the copper-binding sites of CopI confirm the preferential Cu­(II) binding observed for the full-length protein, with the N-terminal region acting as the dominant Cu­(II)-binding site and the internal sequence serving as a secondary, lower-affinity interaction site.

**Figure 10:**
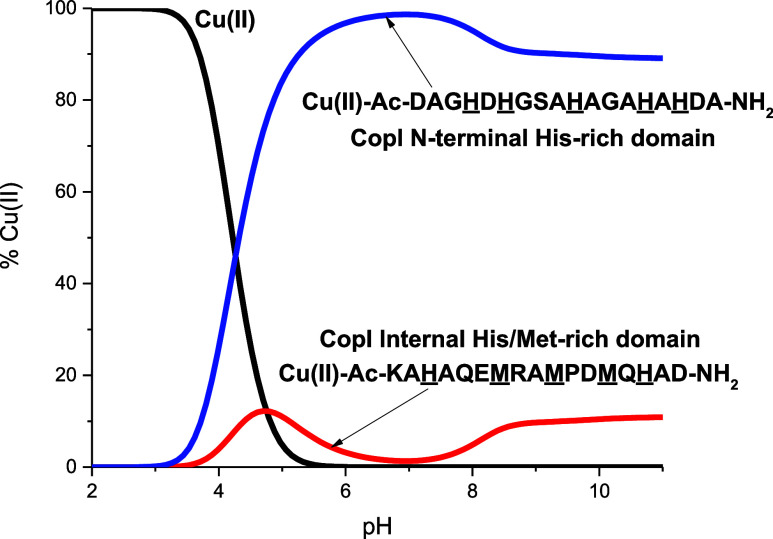
Competition plots for a simulated solution containing equimolar concentrations of Cu­(II), Ac-KAHAQEMRAMPDMQHAD-NH_2_ and Ac-DAGHDHGSAHAGAHAHDA-NH_2_. The diagram is calculated from the overall stability constants of the binary complexes and simulate a situation in which equimolar concentrations of all of the chosen ligands and metal are present in a solution.

#### Zinc­(II) Binding as a Counterpart to Copper­(II) Interaction in the His/Met-Rich Region and N-Terminal His-Rich Domain of CopI

Although CopI is known as a copper-binding protein in bacteria, there is currently no experimental evidence that its binding domains are capable of coordinating Zn­(II). Nevertheless, the chemical features of this segments, particularly the presence of histidine and methionine residues, raise the question of whether this domains might, at least in principle, interact with zinc under certain conditions. For this reason, we aimed to explore the potential Zn­(II) binding ability of the internal His/Met-rich metal-binding motif (Ac-KAHAQEMRAMPDMQHAD-NH_2_) and N-terminal His-rich fragment (Ac-DAGHDHGSAHAGAHAHDA-NH_2_) using a bioinorganic chemistry approach, focusing on their coordination preferences, structural constraints, and possible competition with copper. While CopI functions physiologically as a copper-related protein, understanding how it behaves toward Zn­(II) may shed light on broader aspects of metal selectivity, the structural flexibility of this domains, and the general principles governing metal-protein recognition in metallochaperones.

In this context, we focused specifically on mass spectrometry, potentiometric titrations, and NMR spectroscopy to probe zinc binding, as Zn­(II) is generally spectroscopically silent and does not allow for straightforward characterization by many electronic spectroscopic methods typically used for transition metals. Complementary circular dichroism (CD) measurements in the 180–300 nm range were also performed to assess the structural features of the free ligands and their Zn­(II) complexes, providing additional insight into possible secondary structure induction and conformational changes upon metal coordination.

Similarly to the Cu­(II) complexes, both the internal His/Met-rich and the N-terminal His-rich domains form Zn­(II) complexes with a 1:1 stoichiometry. The average *m*/*z* values for the species observed in the ESI-MS spectra of the Zn­(II) complexes with the Ac-KAHAQEMRAMPDMQHAD-NH_2_ and Ac-DAGHDHGSAHAGAHAHDA-NH_2_ peptides are summarized in Table S3, while the corresponding mass spectra are presented in Figure S9.

For the internal His/Met domain of CopI, the potentiometric data are not fully satisfactory. The first complex species that can be reliably fitted to the experimental data is the ZnHL species, in which two histidine residues are already deprotonated. Two additional species, ZnH_–1_L and ZnH_–2_L, were also calculated; the first species suggests the deprotonation of a coordinated water molecule, which may complement the Zn­(II) binding site, while the second species, with a p*K*_a_ value of 9.91, may indicate the involvement of a second water molecule or result from the deprotonation of an unbound Lys residue. (Figure S10A and Table 5A). Observation of any complex species formed at lower pH values is therefore not feasible. Although Hyperquad calculations indicate that such low-pH species may be present, they would occur only at very low concentrations, below the detection threshold of the program. These results suggest that the internal His/Met domain forms very weak Zn­(II) complexes.

**Table 5:** Stability constants (logβ) for (A) Zn­(II)-Ac-KAHAQEMRAMPDMQHAD-NH_2_ and (B) Zn­(II)-Ac-DAGHDHGSAHAGAHAHDA-NH_2_ complexes in aqueous solution of 4 mM HClO_4_ with *I* = 0.1 M NaClO_4_ at 25 °C^a^

species	logβ	p*K*_a_
Zn(II)-Ac-KAHAQEMRAMPDMQHAD-NH_2_		
ZnHL	13.94(2)	
ZnH_–1_L	–2.66(3)	
ZnH_–2_L	–12.57(5)	9.91
Zn(II)-Ac-DAGHDHGSAHAGAHAHDA-NH_2_		
ZnH_5_L	35.44(4)	
ZnH_4_L	30.79(2)	4.65
ZnH_2_L	19.92(1)	
ZnHL	13.97(1)	5.95
ZnL	7.45(1)	6.52
ZnH_–1_L	–1.13(2)	8.58
ZnH_–2_L	–10.35(2)	9.22

a M/L molar ratio = 0.9:1.

The 1D ^1^H NMR spectrum obtained upon addition of 0.9 equiv of Zn­(II) is practically analogous to that of the apo Ac-KAHAQEMRAMPDMQHAD-NH_2_ peptide (Figure S11). The only significant spectral change is the selective downfield shift observed for the Hε1 imidazole protons of both His3 and His15 residues. This phenomenon is consistent with the electronic deshielding effect induced by metal coordination: the binding of the zinc ion to the imidazole nitrogen withdraws electron density from the aromatic ring, thereby reducing the shielding of the ring protons. Furthermore, the small magnitude of the shift (+0.03 ppm) and the preservation of line sharpness suggest that the system is in a fast exchange regime on the NMR time scale, involving the neutral imidazole side-chains without deprotonation. Consequently, the lack of variation in the remaining signals confirms a specific interaction localized at the histidine side-chains without inducing widespread conformational perturbations.

For the second ligand, the N-terminal His-rich domain of CopI, the first species, ZnH_5_L, likely involves zinc binding to the carboxyl groups of glutamic acid residues (Figure S10B, and Table 5B). The appearance of the next four species, ZnH_4_L, ZnH_2_L, ZnHL and ZnL, with notably shifted p*K*_a_ values (4.65, 5.95, and 6.53 vs 5.47, 6.97, and 7.51 for free ligand), indicates participation of histidine side chains in Zn­(II) binding. It is difficult to estimate how many histidines are involved in Zn­(II) coordination, because all calculated p*K*_a_ values for these species are lower compared to their apo forms. Although it is unlikely that all five histidines bind Zn­(II) simultaneously, some dynamic exchange among the imidazole groups is expected. The last two species, ZnH_–1_L and ZnH_–2_L, with p*K*_a_ values of 8.58 and 9.22, correspond to the deprotonation of a bound water molecules. Taking into account that Zn­(II) typically prefers tetrahedral or trigonal-bipyramidal coordination geometry, it is highly probable that two or three histidines are coordinated at the same time, with two water molecules completing the Zn­(II) coordination sphere.

In contrast to the internal His/Met domain of CopI, the Ac-DAGHDHGSAHAGAHAHDA-NH_2_ peptide exhibits significantly higher reactivity toward the addition of zinc ions, as demonstrated by NMR spectroscopy. This is readily apparent from the inspection of the aromatic region in the 1D NMR spectra during zinc titration (Figure S12). Already at the addition of 0.3 equiv, evident line broadening is observed for the aromatic protons of all five histidine residues, affecting both the Hε1 and Hδ2 signals. Upon reaching 0.9 equiv, these signals undergo almost complete disappearance. The system was further characterized by TOCSY experiments at both 0.3 and 0.9 equiv (Figure S13). As previously mentioned, due to the overlap of analogous signals, unambiguous assignment of specific residues for repeated amino acids was not possible. At 0.9 equiv, the presence of zinc alters the correlations of at least four out of the six alanines, resulting in total line broadening. Regarding the aspartates, two out of the three residues undergo complete broadening of the NH-Hβ correlation. Unlike the overlapped residues, Ser8 residue is clearly distinguishable; at 0.9 equiv of Zn­(II), it exhibits evident broadening in both the NH-Hα and NH-Hβ correlations. Furthermore, the three glycine residues display a generalized broadening of their NH-Hα cross-peaks. Collectively, these NMR findings indicate that the system is in an intermediate exchange regime on the NMR time scale. This suggests that zinc binding induces a significant conformational rearrangement, likely a folding event, where the peptide interconverts between free and bound forms (or different folded conformers) with kinetics comparable to the chemical shift time scale, resulting in the observed loss of signal intensity.

In structural circular dichroism (CD) analyses, the addition of Zn­(II) to the internal His/Met domain of CopI (Ac-KAHAQEMRAMPDMQHAD-NH_2_) does not induce any detectable changes across the entire pH range (Figure S14A), and the resulting CD spectrum remains identical to that of the apo form (Figure 4A). In contrast, Zn­(II) binding to the N-terminal His-rich domain (Ac-DAGHDHGSAHAGAHAHDA-NH_2_) (Figure S14B) gives rise to the same spectral alterations as those observed for the Cu­(II) complexes (Figure 7B), and differs from the apo form, particularly at pH values above 7, where coordination to imidazole nitrogens becomes evident.

## Conclusions

In this work, we show that the copper tolerance protein CopI contains functionally distinct metal-binding motifs that differ markedly in coordination chemistry and metal affinity. Using model peptides representing individual CopI regions, we demonstrate that both the N-terminal histidine-rich domain and the internal histidine/methionine-rich motif form stable mononuclear 1:1 complexes with Cu­(II), yet the resulting coordination environments are clearly different.

At pH 7.4, the N-terminal His-rich peptide stabilizes Cu­(II) mainly through a nitrogen-donor environment. Spectroscopic and potentiometric data support a coordination mode involving two histidine imidazole nitrogens together with a deprotonated backbone amide nitrogen. NMR data further indicate exchange among multiple histidine residues, pointing to coordination dynamics around a stable Cu­(II) core geometry. This combination of a N-dominated donor set and limited structural plasticity is consistent with the higher Cu­(II) affinity of the N-terminal region. By contrast, the internal His/Met-rich peptide exhibits a more heterogeneous coordination environment under physiological conditions. While Cu­(II) binding remains centered on two histidine imidazole nitrogens, NMR data reveal additional contributions from at least one methionine thioether sulfur and carboxylate oxygen donors from aspartate residues. This mixed N/O/S donor set leads to a less rigid coordination sphere and reduced overall Cu­(II) stability, consistent with a motif optimized for adaptability rather than maximal binding strength. The observed differences in Cu­(II) coordination reflect a division of labor between CopI domains that is well suited to the redox-dynamic conditions of the bacterial periplasm.

The N-terminal domain displays higher Cu­(II) affinity and predominantly histidine-based, nitrogen-donor coordination, features that are consistent with a potential copper-buffering or sequestration role. In contrast, the internal His/Met-rich motif binds Cu­(II) more weakly and supports a more heterogeneous and adaptable donor set. Such coordination flexibility may be compatible with interactions involving reduced Cu­(I) species, which preferentially bind softer donor atoms such as sulfur. However, this possibility remains indirect in the present work, since Cu­(I) binding was not examined experimentally owing to the limited suitability of potentiometric analysis for methionine-containing Cu­(I) systems and the instability of Cu­(I) in histidine-rich peptide environments under titration conditions. Importantly, the comparatively weaker Cu­(II) affinity of the His/Met-rich motif should therefore not be interpreted as a deficiency, but rather as an intrinsic chemical feature of this sequence environment. Instead, the observed differences between the two motifs suggest that they may fulfill complementary roles in copper binding and handling within CopI. It should be noted, however, that the present peptide-based approach isolates the intrinsic coordination properties of these motifs and therefore does not directly address the mechanistic role of CopI in vivo. Consequently, the functional implications proposed here should be regarded as hypotheses that require verification in the context of the intact protein. Within this framework, Cu­(II) coordination provides a useful reference state for highlighting the chemical asymmetry between the two CopI domains.

The absence of stable secondary structure upon metal binding observed for both investigated domains suggests that these motifs retain a high degree of conformational flexibility. Such structural plasticity may facilitate ligand exchange and dynamic metal coordination. Interestingly, NMR studies on the full-length CopI protein indicate that the central His/Met-rich region exhibits dynamic behavior and interacts with the cupredoxin domain,^8^ which is consistent with the flexible coordination properties observed for the isolated motifs. However, because the present experiments were performed on isolated peptide fragments, the functional implications for the full-length CopI protein remain tentative.

Although CopI is copper-specialized, comparative Zn­(II) binding experiments provide a coordination-chemical reference for a redox-inactive metal ion. This observation is consistent with general coordination-chemical principles, including the Irving–Williams series, which predicts higher thermodynamic stability for Cu­(II) complexes relative to Zn­(II). Both CopI-derived motifs bind Zn­(II) more weakly than Cu­(II), with coordination dominated by histidine donors and without the heterogeneity and dynamic features observed for the copper complexes. This contrast highlights that CopI is tuned for copper handling rather than general metal sequestration.

Taken together, these findings indicate that CopI exploits modular coordination strategies to stabilize copper differently across its domains. The coexistence of a rigid, nitrogen-dominated site and a flexible His/Met-rich environment may allow CopI to buffer copper redox chemistry and contribute to copper tolerance in the bacterial periplasm under microaerobic conditions. This work establishes CopI as a unique example of a green cupredoxin that exploits intrinsic disorder and modular coordination chemistry to manage copper stress in the bacterial periplasm.

## Supplementary Material



## Notes

High-resolution ESI-MS spectrum; pH-dependent UV-vis absorption spectra; X-band EPR spectra of frozen solution; CD spectra (PDF)
