# Supplementary material for: The Two Faces of Copper Capture Driven by the Internal His/Met-Rich and N‑Terminal His-Rich Domains of CopI
Source: Inorg Chem. 2026 Apr 24;65(18):10121–34. doi: 10.1021/acs.inorgchem.6c00726 (PMC13308877; doi:10.1021/acs.inorgchem.6c00726)
Supplement: Supplementary file 1 [file ic6c00726_si_001.pdf]

# Supporting Information

## The two faces of copper capture driven by the internal His/Met-rich and N-terminal His-rich domains of CopI

Paulina Sobol,[1] Arian Kola,[2] Daniela Valensin,[2,3] Aleksandra Hecel[1]*

[1] Faculty of Chemistry, University of Wroclaw, 50383 Wroclaw, Poland.

[2] Department of Biotechnology, Chemistry and Pharmacy, University of Siena, 53100 Siena, Italy.

[3] Consorzio Interuniversitario Risonanze Magnetiche di Metalloproteine (CIRMMP), Via L. Sacconi 6, Sesto Fiorentino 50019, Italy

*aleksandra.hecel2@uwr.edu.pl

**Table S1** Stoichiometry, molecular formula and average m/z value for the species present in ESI-MS spectra of Cu(II) complexes with the Ac-KAHAQEMRAMPDMQHAD-$NH_2$, M:L molar ratio = 0.9:1 in water:methanol 50:50 solution.

| m/z | ion | Molecular formula |
|---|---|---|
| 1004.91 | $[L]^{2+}$ | $C_{81}H_{130}N_{28}O_{26}S_3$ |
| 1015.90 | $[L+Na]^{2+}$ | $C_{81}H_{129}N_{28}O_{26}S_3Na$ |
| 1023.89 | $[L+K]^{2+}$ | $C_{81}H_{129}N_{28}O_{26}S_3K$ |
| 1026.89 | $[L+2Na]^{2+}$ | $C_{81}H_{128}N_{28}O_{26}S_3Na_2$ |
| 1046.85 | $[L+3Na]^{2+}$ | $C_{81}H_{127}N_{28}O_{26}S_3Na_2$ |
| 1034.88 | $[CuL]^{2+}$ | $C_{81}H_{127}N_{28}O_{26}S_3Cu$ |
| 670.27 | $[L]^{3+}$ | $C_{81}H_{130}N_{28}O_{26}S_3$ |
| 677.60 | $[L+Na]^{3+}$ | $C_{81}H_{129}N_{28}O_{26}S_3Na$ |
| 682.92 | $[L+K]^{3+}$ | $C_{81}H_{129}N_{28}O_{26}S_3K$ |
| 684.93 | $[L+2Na]^{3+}$ | $C_{81}H_{128}N_{28}O_{26}S_3Na_2$ |
| 690.25 | $[CuL]^{3+}$ | $C_{81}H_{127}N_{28}O_{26}S_3Cu$ |
| 697.57 | $[CuL+Na]^{3+}$ | $C_{81}H_{126}N_{28}O_{26}S_3CuNa$ |
| 705.56 | $[CuL+2Na]^{3+}$ | $C_{81}H_{126}N_{28}O_{26}S_3CuNa_2$ |
| 502.96 | $[L]^{4+}$ | $C_{81}H_{130}N_{28}O_{26}S_3$ |
| 508.45 | $[L+Na]^{4+}$ | $C_{81}H_{129}N_{28}O_{26}S_3Na$ |
| 512.44 | $[L+K]^{4+}$ | $C_{81}H_{129}N_{28}O_{26}S_3K$ |
| 513.95 | $[L+2Na]^{4+}$ | $C_{81}H_{128}N_{28}O_{26}S_3Na_2$ |
| 519.44 | $[L+3Na]^{4+}$ | $C_{81}H_{127}N_{28}O_{26}S_3Na_2$ |
| 517.94 | $[CuL]^{4+}$ | $C_{81}H_{127}N_{28}O_{26}S_3Cu$ |

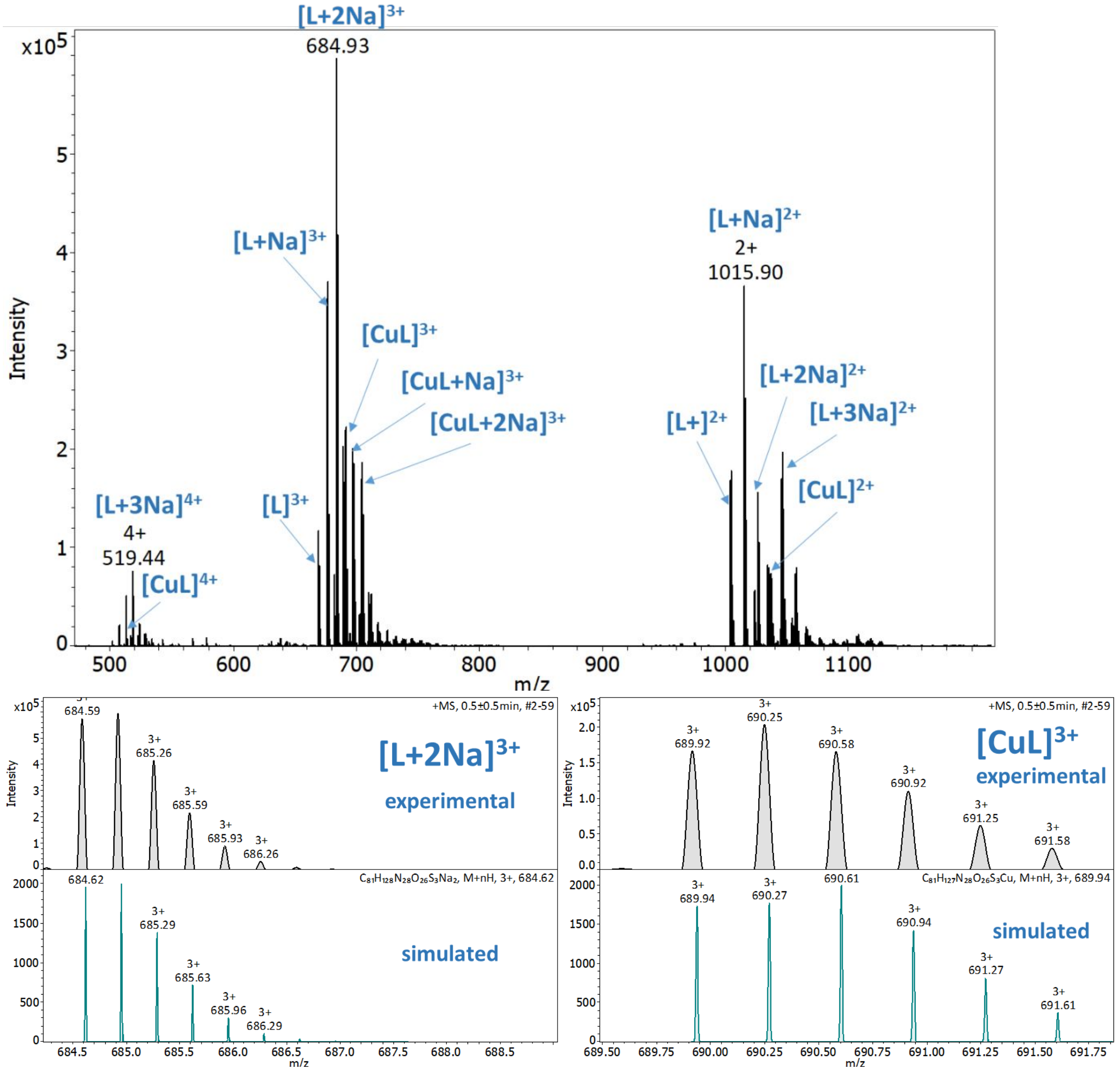


**Figure S1** High-resolution ESI-MS spectrum of Cu(II) complexes with the Ac-KAHAQEMRAMPDMQHAD-$NH_2$, M:L molar ratio = 0.9:1 in water:methanol 50:50 solution. Below: comparison of experimental and simulated isotopic patterns for $[L+2Na]^{3+}$, and $[CuL]^{3+}$ species.

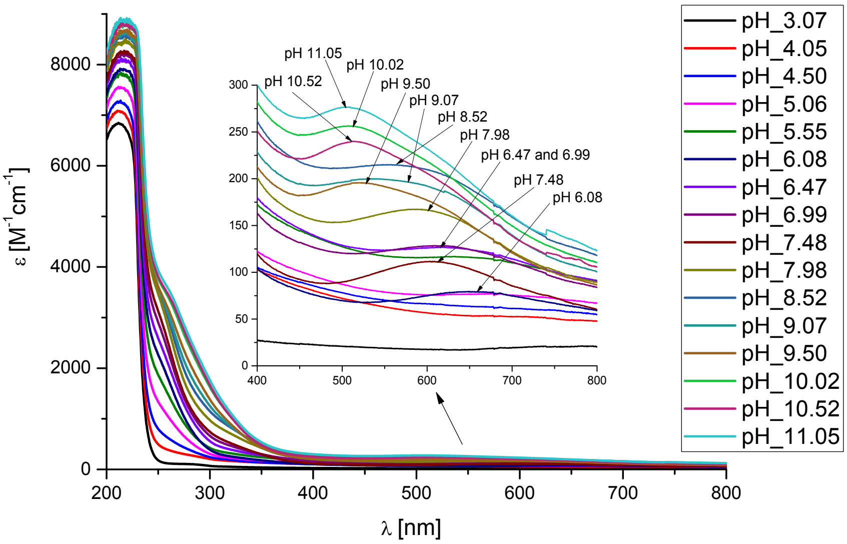


**Figure S1** pH-dependent UV-vis absorption spectra for Cu(II)-Ac-KAHAQEMRAMPDMQHAD-$NH_2$ in aqueous solution of 4 mM $HClO_4$ with I = 0.1 M $NaClO_4$, T = 298 K, M:L molar ratio = 0.9:1, $C_L$ = 0.4 mM.

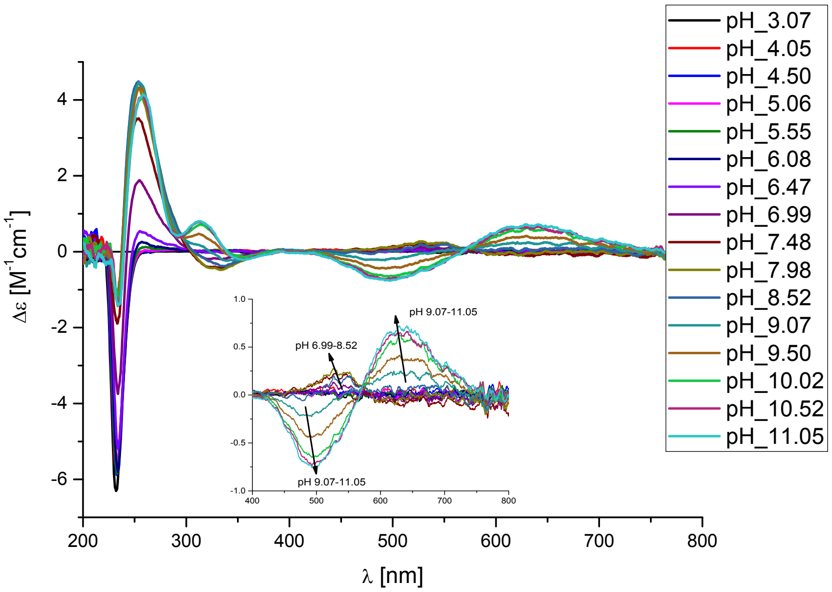


**Figure S2** pH-dependent CD spectra for Cu(II)-Ac-KAHAQEMRAMPDMQHAD-$NH_2$ in aqueous solution of 4 mM $HClO_4$ with I = 0.1 M $NaClO_4$, T = 298 K, M:L molar ratio = 0.9:1, $C_L$ = 0.4 mM.

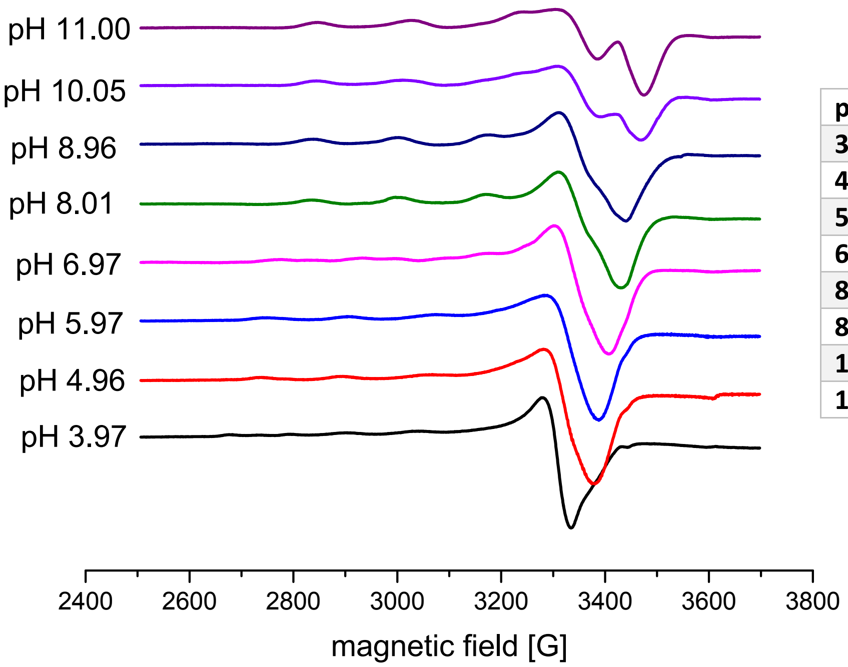


| **pH** | **$A_{II}$** | **$g_{II}$** |
|---|---|---|
| **3.97** | 118 | 2.42 |
| **4.96** | 157 | 2.32 |
| **5.97** | 163 | 2.29 |
| **6.97** | 164 | 2.27 |
| **8.01** | 170 | 2.22 |
| **8.96** | 170 | 2.22 |
| **10.05** | 180 | 2.20 |
| **11.00** | 185 | 2.19 |

**Figure S4** X-band EPR spectra of frozen solution (77 K) of Cu(II)-Ac-KAHAQEMRAMPDMQHAD-$NH_2$ at different pH values; I=0.1 M ($NaClO_4$), metal:ligand molar ratio 0.9:1, [Cu(II)] = 1 mM. EPR parameters $A_{II}$ and $g_{II}$ for different pH values corresponding to the maximum concentrations of given complex species are collected at Table 2 in the main text.

**Table S2** Stoichiometry, molecular formula and average m/z value for the species present in ESI-MS spectra of Cu(II) complexes with the Ac-DAGHDHGSAHAGAHAHDA-$NH_2$, M:L molar ratio = 0.9:1 in water:methanol 50:50 solution.

| m/z | ion | Molecular formula |
|---|---|---|
| 887.89 | $[L]^{2+}$ | $C_{71}H_{99}N_{29}O_{26}$ |
| 898.88 | $[L+Na]^{2+}$ | $C_{71}H_{98}N_{29}O_{26}Na$ |
| 909.86 | $[L+2Na]^{2+}$ | $C_{71}H_{97}N_{29}O_{26}Na_2$ |
| 918.34 | $[CuL]^{2+}$ | $C_{71}H_{97}N_{29}O_{26}Cu$ |
| 929.33 | $[CuL+Na]^{2+}$ | $C_{71}H_{96}N_{29}O_{26}CuNa$ |
| 940.32 | $[CuL+2Na]^{2+}$ | $C_{71}H_{95}N_{29}O_{26}CuNa_2$ |
| 592.27 | $[L]^{3+}$ | $C_{71}H_{99}N_{29}O_{26}$ |
| 599.81 | $[L+Na]^{3+}$ | $C_{71}H_{98}N_{29}O_{26}Na$ |
| 606.92 | $[L+2Na]^{3+}$ | $C_{71}H_{97}N_{29}O_{26}Na_2$ |
| 612.58 | $[CuL]^{3+}$ | $C_{71}H_{97}N_{29}O_{26}Cu$ |
| 619.90 | $[CuL+Na]^{3+}$ | $C_{71}H_{96}N_{29}O_{26}CuNa$ |
| 627.23 | $[CuL+2Na]^{3+}$ | $C_{71}H_{95}N_{29}O_{26}CuNa_2$ |

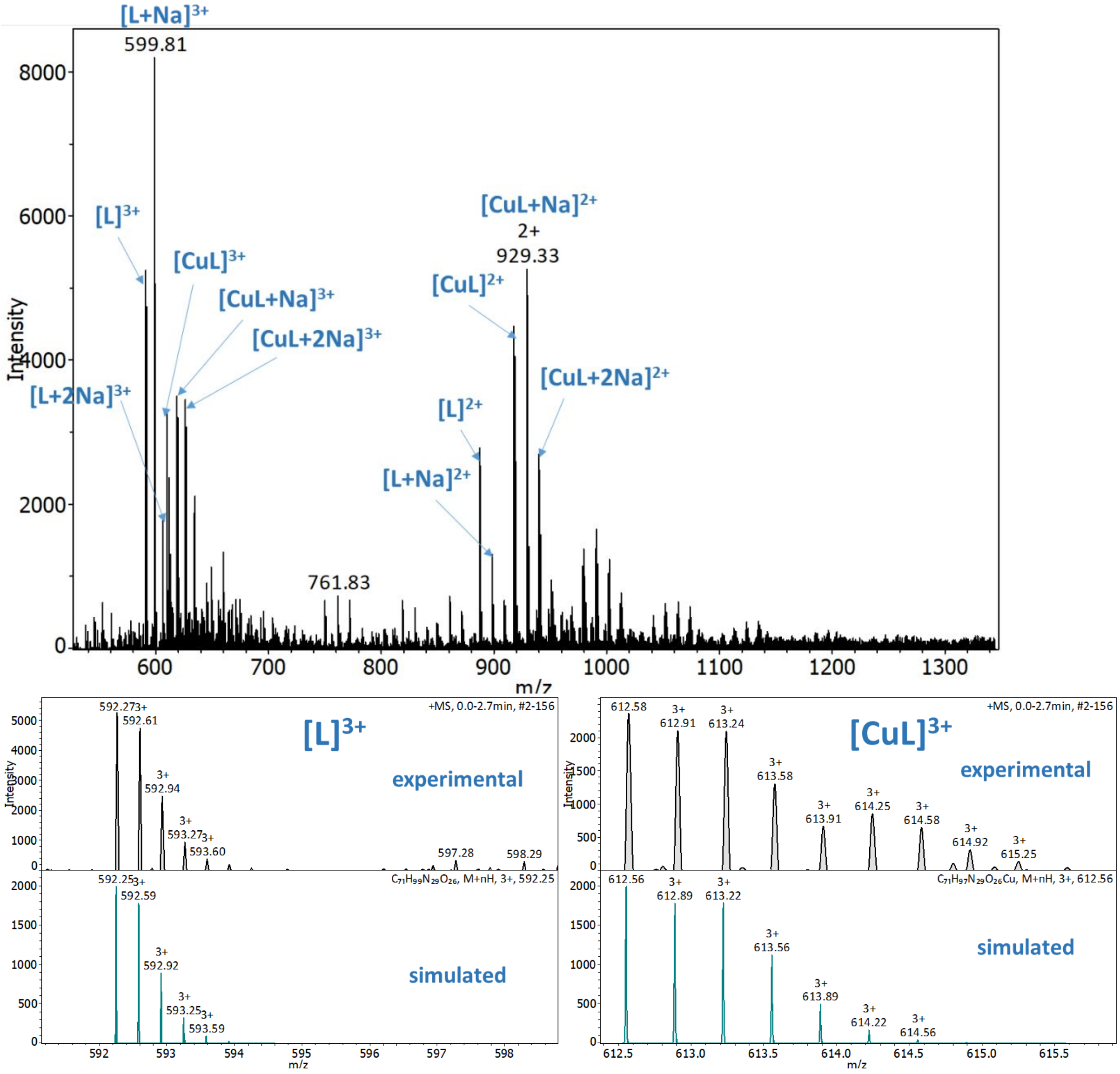


**Figure S5** High-resolution ESI-MS spectrum of Cu(II) complexes with the Ac-DAGHDHGSAHAGAHAHDA-$NH_2$, M:L molar ratio = 0.9:1 in water:methanol 50:50 solution. Below: comparison of experimental and simulated isotopic patterns for $[L]^{3+}$, and $[CuL]^{3+}$ species.

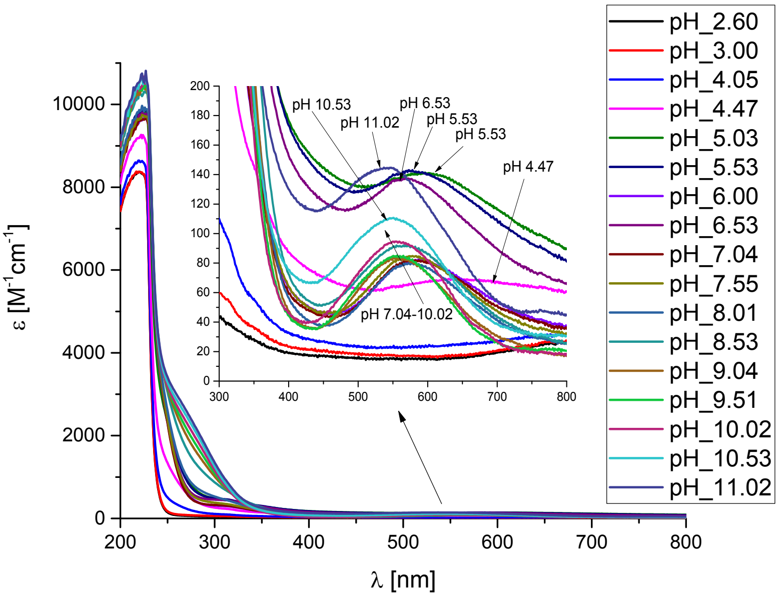


**Figure S6** pH-dependent UV-vis absorption spectra for Cu(II)-Ac-DAGHDHGSAHAGAHAHDA-$NH_2$ in aqueous solution of 4 mM $HClO_4$ with I = 0.1 M $NaClO_4$, T = 298 K, M:L molar ratio = 0.9:1, $C_L$ = 0.4 mM.

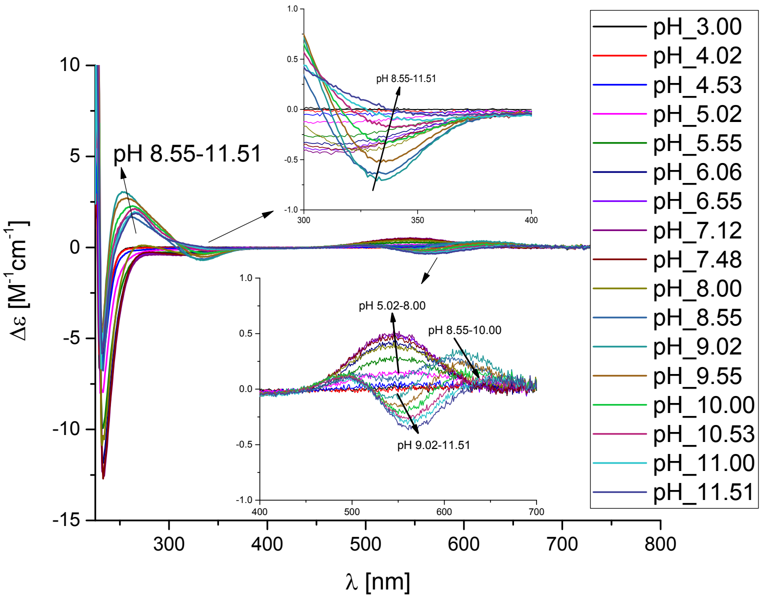


**Figure S7** pH-dependent CD spectra for Cu(II)-Ac-DAGHDHGSAHAGAHAHDA-$NH_2$ in aqueous solution of 4 mM $HClO_4$ with I = 0.1 M $NaClO_4$, T = 298 K, M:L molar ratio = 0.9:1, $C_L$ = 0.4 mM.

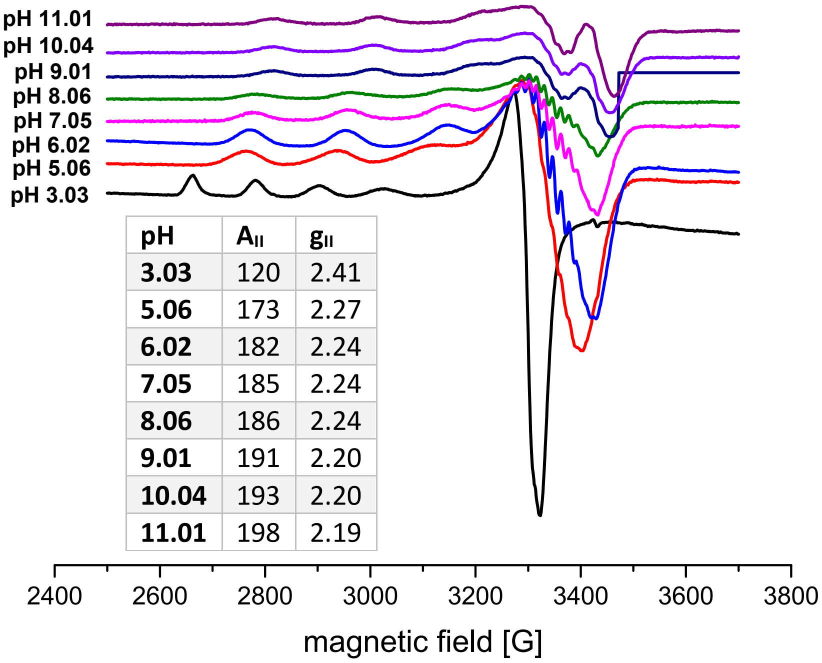


**Figure S8** X-band EPR spectra of frozen solution (77 K) of Cu(II)-Ac-DAGHDHGSAHAGAHAHDA-$NH_2$ at different pH values; I=0.1 M ($NaClO_4$), metal:ligand molar ratio 0.9:1, [Cu(II)] = 1 mM. EPR parameters $A_{II}$ and $g_{II}$ for different pH values corresponding to the maximum concentrations of given complex species are collected at Table 4 in the main text.

**Table S3** Stoichiometry, molecular formula and average m/z value for the species present in ESI-MS spectra of Zn(II) complexes with the A) Ac-KAHAQEMRAMPDMQHAD-$NH_2$ and B) Ac-DAGHDHGSAHAGAHAHDA-$NH_2$, M:L molar ratio = 0.9:1 in water:methanol 50:50 solution. Values for differently charged ligand species are omitted, they are provided in Tables S1 and S2.

| m/z | ion | Molecular formula |
|---|---|---|
| **A) Zn(II)-Ac-KAHAQEMRAMPDMQHAD-$NH_2$** | | |
| 1035.43 | $[ZnL]^{+2}$ | $C_{81}H_{126}N_{28}O_{26}S_3Zn$ |
| 1046.42 | $[ZnL+Na]^{+2}$ | $C_{81}H_{125}N_{28}O_{26}S_3ZnNa$ |
| 690.62 | $[ZnL]^{+3}$ | $C_{81}H_{126}N_{28}O_{26}S_3Zn$ |
| 697.96 | $[ZnL+Na]^{+3}$ | $C_{81}H_{125}N_{28}O_{26}S_3ZnNa$ |
| 705.28 | $[ZnL+2Na]^{+3}$ | $C_{81}H_{124}N_{28}O_{26}S_3ZnNa_2$ |
| 518.24 | $[ZnL]^{+4}$ | $C_{81}H_{126}N_{28}O_{26}S_3Zn$ |
| 523.73 | $[ZnL+Na]^{+3}$ | $C_{81}H_{125}N_{28}O_{26}S_3ZnNa$ |
| **B) Zn(II)-Ac-DAGHDHGSAHAGAHAHDA-$NH_2$** | | |
| 919.84 | $[ZnL]^{+2}$ | $C_{71}H_{97}N_{29}O_{26}Zn$ |
| 930.83 | $[ZnL+Na]^{+2}$ | $C_{71}H_{96}N_{29}O_{26}ZnNa$ |
| 941.82 | $[ZnL+2Na]^{+2}$ | $C_{71}H_{95}N_{29}O_{26}ZnNa_2$ |
| 952.81 | $[ZnL+3Na]^{+2}$ | $C_{71}H_{94}N_{29}O_{26}ZnNa_3$ |
| 628.23 | $[ZnL+2Na]^{+3}$ | $C_{71}H_{95}N_{29}O_{26}ZnNa_2$ |

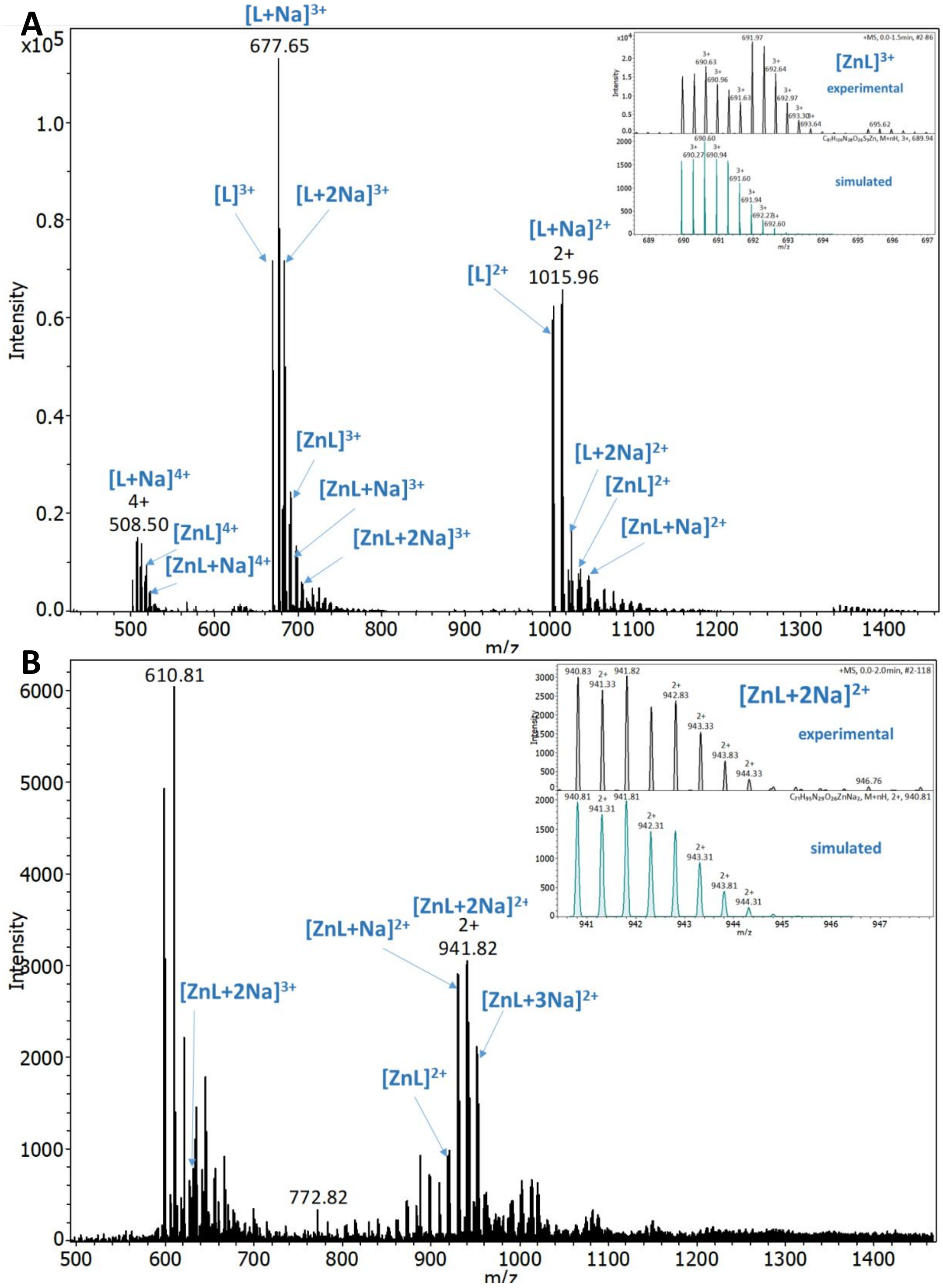


**Figure S9** High-resolution ESI-MS spectra of Zn(II) complexes with (A) the Ac-KAHAQEMRAMPDMQHAD-$NH_2$ with comparison of experimental and simulated isotopic patterns for $[ZnL]^{3+}$ species; (B) the Ac-DAGHDHGSAHAGAHAHDA-$NH_2$ with comparison of experimental and simulated isotopic patterns for $[ZnL+2Na]^{2+}$ species. M:L molar ratio = 0.9:1 in water:methanol 50:50 solution.

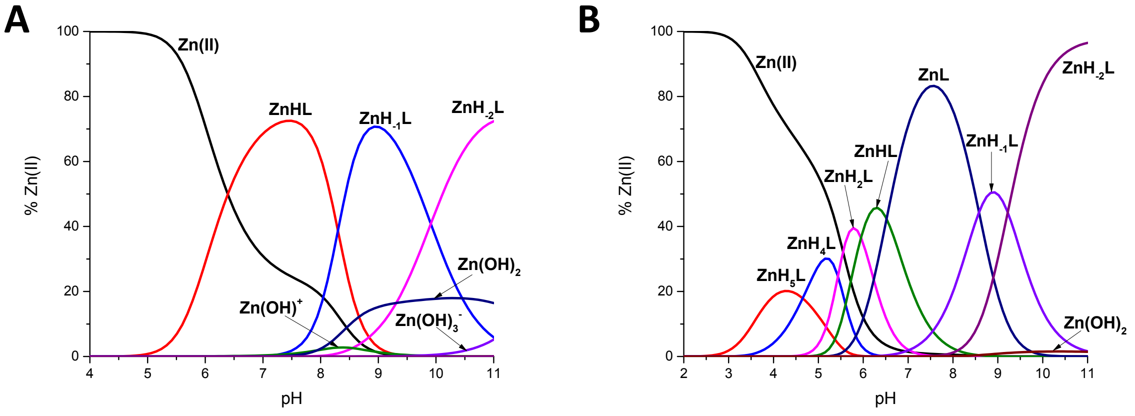


**Figure S10** Distribution diagrams for the formation of Zn(II) complex with A) Ac-KAHAQEMRAMPDMQHAD-$NH_2$ and B) Ac-DAGHDHGSAHAGAHAHDA-$NH_2$, T = 298 K and I = 0.1 M ($NaClO_4$), M:L molar ratio = 0.9:1, $C_L$ = 0.4 mM.

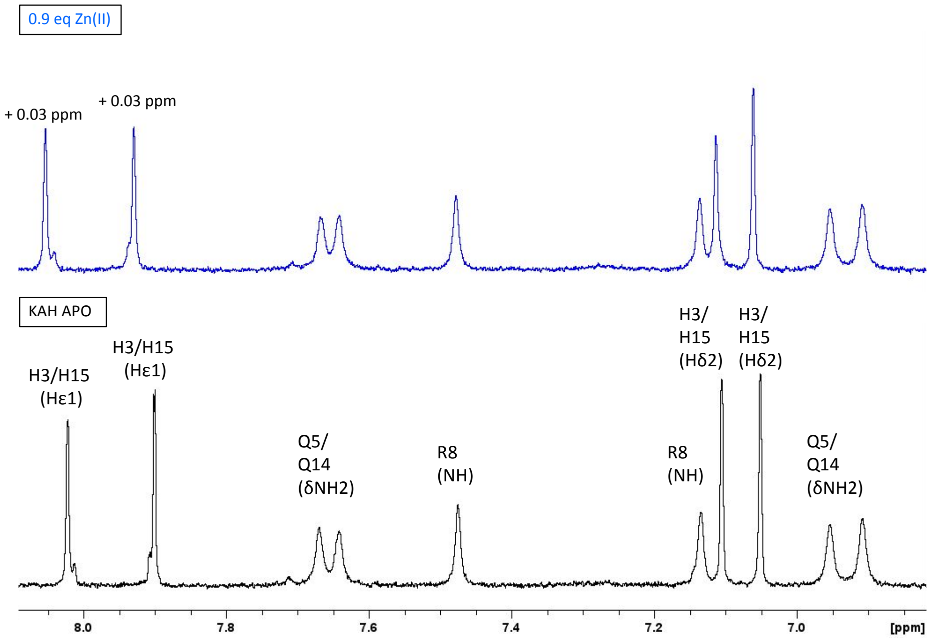


**Figure S11** Superimposition of the 1D spectra of Ac-KAHAQEMRAMPDMQHAD-$NH_2$ recorded in absence and in presence of 0.9 Zn(II) eqs. T=288 K, $C_M$=0.5 mM, phosphate buffer=20 mM, pH 7.4.

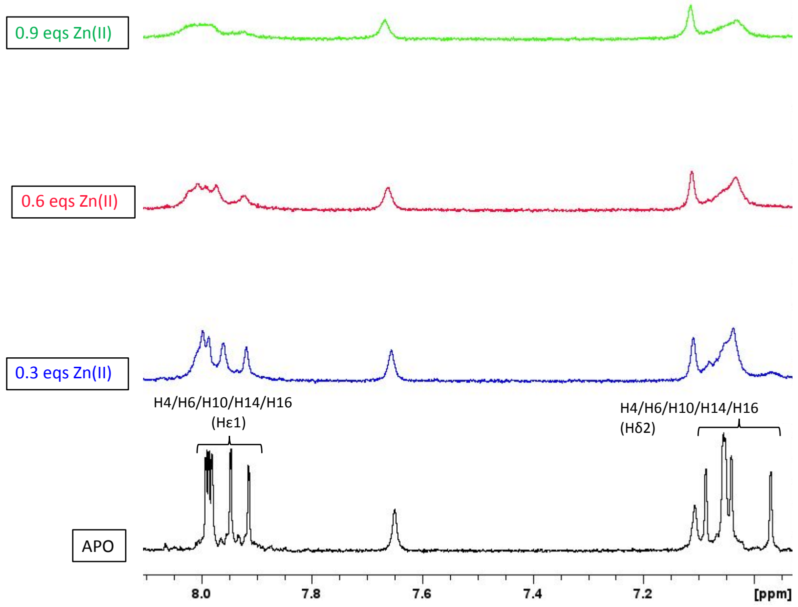


**Figure S12** Superimposition of the 1D spectra of Ac-DAGHDHGSAHAGAHAHDA-$NH_2$ recorded in absence and in presence of 0.3, 0.6 and 0.9 Zn(II) eqs. T=288 K, $C_M$=0.5 mM, phosphate buffer=20 mM, pH 7.4.

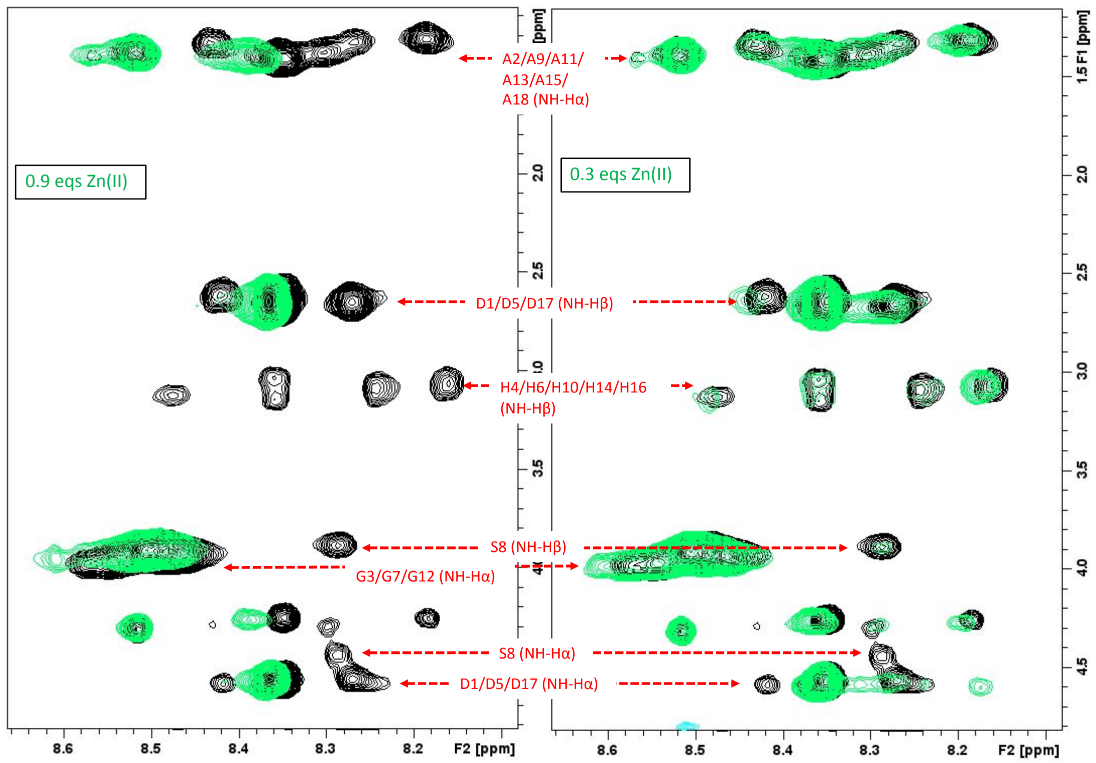


**Figure S13** Superimposition of the (A,B) finger print and (C) aromatic regions of 2D $^1H$-$^1H$ TOCSY spectra of Ac-DAGHDHGSAHAGAHAHDA-$NH_2$ recorded in absence (black) and in presence of 0.3 (left) and 0.9 (right) Zn(II) eqs. (green). T=288 K, $C_M$=0.5 mM, phosphate buffer=20 mM, pH 7.4.

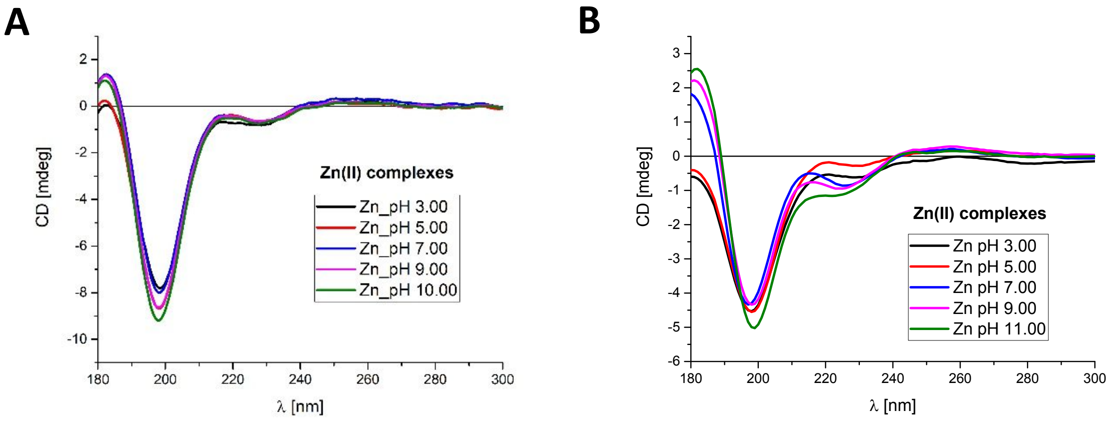


**Figure S14** CD spectra for A) Zn(II)-Ac-KAHAQEMRAMPDMQHAD-$NH_2$ B) Zn(II)- Ac-DAGHDHGSAHAGAHAHDA-$NH_2$ in aqueous solution of 4 mM $HClO_4$ with I = 0.1 M $NaClO_4$, T = 298 K, M:L molar ratio = 0.9:1, $C_L$ = 0.1 mM.
